# Edentulism and functional disability in older adults: A longitudinal and cross-national study in England, the United States, and India

**DOI:** 10.1371/journal.pone.0361422

**Published:** 2026-10-30

**Authors:** Wanqi Tang, Libang He, Min Zhang, Jiyao Li

**Affiliations:** State Key Laboratory of Oral Diseases, National Clinical Research Center for Oral Diseases, Department of Cariology and Endodontics, West China Hospital of Stomatology, Sichuan University, Chengdu, Sichuan, China; Fondazione Don Carlo Gnocchi, ITALY

## Abstract

Edentulism may contribute to functional disability in older adults, yet cross-national evidence combining multiple analytic strategies and mechanistic assessment remains limited. This study examined the association between edentulism and disability in activities of daily living (ADL) and instrumental activities of daily living (IADL) across England, the United States, and India. We analyzed harmonized data from the English Longitudinal Study of Ageing (ELSA; n = 9,102), the Health and Retirement Study (HRS; n = 22,156), and the Longitudinal Ageing Study in India (LASI; n = 31,816) for adults aged 60 years or older. Cross-sectional associations were estimated using inverse probability weighting (IPW) and augmented IPW. Longitudinal Cox proportional hazards models were applied in ELSA and HRS. Mediation through grip strength, body mass index (BMI), and depressive symptoms was assessed with counterfactual causal mediation analysis on the risk-difference scale. Edentulism was associated with higher odds of ADL (pooled odds ratio 1.30, 95% confidence interval 1.20–1.42) and IADL disability (1.27, 1.11–1.45). Longitudinal analyses confirmed elevated hazard ratios for incident ADL (1.19, 1.09–1.30) and IADL disability (1.30, 1.19–1.43). Grip strength accounted for the largest proportion mediated (approximately 50% in ELSA and HRS, and 5–14% in LASI); BMI and depressive symptoms showed little mediation. In LASI, denture use among edentulous participants was associated with lower disability odds. Edentulism was consistently associated with functional disability across three countries, with grip strength partially accounting for this association, although the cross-sectional design precludes establishing temporality. Oral rehabilitation may partly buffer this risk and merits further prospective investigation.

## Introduction

Edentulism remains one of the most consequential manifestations of oral disease in later life. Although the global burden of oral disease has shifted over time, complete tooth loss still marks a substantial loss of oral function and quality of life, particularly in older adults with accumulated social and medical vulnerability [1–4]. In practical terms, edentulism is not simply the absence of teeth. It alters chewing efficiency, narrows food choices, changes speech and appearance, and can gradually weaken social participation and physical reserve.

Functional disability, commonly captured through limitations in activities of daily living (ADL) and instrumental activities of daily living (IADL) [5,6], has direct implications for dependence, institutionalization, and survival. Several cohort studies have reported that tooth loss or poor oral status predicts later functional decline [7–9]. A recent systematic review and meta-analysis likewise concluded that deteriorated oral health and oral function are associated with physical disability and mortality in community-dwelling older adults, but the pooled literature still showed wide heterogeneity in design, exposure definition, and confounder control [10]. Earlier work from the Tsurugaya Project linked lower occlusal force to incident disability and adverse health outcomes, supporting the idea that oral function and whole-body function are connected [11–13].

The likely pathways from edentulism to disability are broader than a simple oral-local explanation. Reduced dentition can impair mastication and nutritional intake, which may accelerate loss of muscle strength and lean mass, a process consistent with the growing literature linking oral health to sarcopenia and grip strength decline [14–17]. A second route is psychosocial: eating difficulties, altered appearance, embarrassment, and social withdrawal may contribute to depressive symptoms, which in turn can worsen mobility and daily function [18,19]. By contrast, purely anthropometric measures such as body mass index (BMI) may capture this process only partially. On the basis of these mechanisms we pre-specified three candidate mediators that are measured comparably across the three cohorts: grip strength, as a marker of systemic muscle strength and physical reserve; body mass index, as a coarse proxy for nutritional status; and depressive symptoms, as a marker of the psychosocial pathway.

Despite these plausible mechanisms, most existing evidence comes from single-country analyses using conventional regression, and few studies have attempted to harmonize individual-level data across diverse ageing cohorts while simultaneously addressing confounding, temporal ordering, and mechanistic pathways. Matsuyama and colleagues used a natural experiment framework in ELSA and reported that tooth loss reduced functional capacity in older adults [7]. The JAGES cohort similarly found that poorer oral status predicted incident functional disability [8]. The meta-analysis by Ko and colleagues synthesized broader evidence but relied on aggregate-level pooling [10]. What is still missing is a coordinated cross-national analysis that asks the same question in more than one social and health-care context using individual-level data, while also moving beyond a single modeling framework.

We therefore used harmonized data from England (ELSA), the United States (HRS), and India (LASI) to estimate the association between edentulism and functional disability with inverse probability weighting, augmented inverse probability weighting, and longitudinal Cox models. We also examined whether grip strength, BMI, and depressive symptoms might explain part of the association, and we used several sensitivity analyses (multiple imputation, survey weighting, and denture status) to test robustness. We hypothesized that edentulism would be associated with higher disability risk across cohorts, with muscle strength emerging as the most consistent pathway.

## Materials and methods

### Study design and data sources

This study used harmonized individual-level data from three population-based ageing cohorts: the English Longitudinal Study of Ageing (ELSA) [20], the Health and Retirement Study (HRS) [21], and the Longitudinal Ageing Study in India (LASI) [22]. For all three cohorts we used the publicly documented Harmonized datasets produced by the Gateway to Global Aging Data initiative, which apply a common variable-derivation protocol across studies [23]. These three cohorts share a common design philosophy and have been the subject of extensive cross-national harmonization efforts [23,24]. For the cross-sectional analysis, we included participants aged 60 years or older who had valid information on edentulism and either ADL or IADL disability. After harmonization and cleaning, the final analytic sample comprised 9,102 ELSA participants, 22,156 HRS participants, and 31,816 LASI participants. The contributing waves were ELSA waves 2–9 (2004–2019), HRS waves 7–15 (2004–2020), and the LASI baseline wave (2017–2018); for the cross-sectional models each participant contributed a single record drawn from the most recent wave with complete outcome data.

To build a single cross-sectional record per participant, we selected the last observation with complete outcome information. Exposure status was taken from the participant’s latest observed edentulism record rather than being inferred solely from covariates. For longitudinal analyses, only ELSA and HRS were eligible because repeated waves were available in a form suitable for follow-up modeling. The longitudinal baseline was defined as the earliest wave at which edentulism was observed and both ADL and IADL disability were absent.

### Variable harmonization

Harmonization followed the Gateway to Global Aging Data protocol, which maps cohort-specific survey items onto a shared set of derived variables with identical names, coding, and units across ELSA, HRS, and LASI [23,24]. We harmonized the exposure (edentulism, derived from each study’s report of having lost all natural teeth), the two disability outcomes (each constructed from five matched ADL and five matched IADL items), the three mediators (grip strength in kilograms, body mass index in kg/m2, and a depressive-symptom indicator), and the full covariate set (age, sex, education, marital status, smoking, drinking, hypertension, diabetes, heart disease, stroke, arthritis, lung disease, cancer, and recent falls). For every variable we confirmed that the response categories and the direction of coding matched across cohorts before pooling; continuous measures were kept on their native scales and binary or ordinal measures were recoded to a common reference category. Where an item was collected under a cohort-specific protocol (for example, single- versus dual-hand grip measurement), we documented the difference and used the maximum available value so that the harmonized variable retained a consistent interpretation across studies.

### Analytic overview

The analysis proceeded in four pre-specified stages, summarized in S4 Fig in S1 File. We first estimated cross-sectional associations using propensity-score weighting (IPW) with a doubly robust check (AIPW); we then tested temporal ordering with longitudinal Cox models in the two cohorts with suitable repeated waves; we next examined candidate mechanisms with mediation analysis; and we finally assessed robustness through a pre-specified set of sensitivity analyses together with a supportive machine-learning comparison. The weighting, longitudinal, and mediation analyses address distinct questions (the magnitude of the association, its temporal ordering, and the mechanisms that may carry it) and are reported as complementary parts of a single triangulation strategy rather than as competing primary analyses.

### Exposure, outcomes, mediators, and covariates

The exposure was edentulism, defined as complete loss of natural teeth. The primary outcomes were binary ADL disability and binary IADL disability, each defined as at least one limitation among five harmonized items. ADL items covered dressing, bathing, eating, getting in or out of bed, and toileting [5]. IADL items covered meal preparation, shopping, telephone use, medication management, and money management [6].

Three mediators were examined. Grip strength was modeled as a continuous measure and was derived from cohort-specific grip assessments; when left- and right-hand measurements were available, the observed grip variable represented the maximum available value. BMI was analyzed as a continuous anthropometric measure. Depressive symptoms were measured with harmonized Center for Epidemiologic Studies Depression (CES-D) symptom-count scales. ELSA and HRS used the eight-item version (range 0–8) [25], whereas LASI used the ten-item version (range 0–10) [26]; in the Gateway to Global Aging Harmonized datasets both are scored as counts of reported depressive symptoms rather than as the summed Likert scores of the original instruments [23]. For cross-cohort comparability we dichotomized each scale at four or more reported symptoms to denote elevated depressive symptoms, a count-based threshold that has been applied to the harmonized eight-item scale in previous cohort analyses [25]. Because the underlying instruments and item counts differ across cohorts, we treated this variable as an approximate, harmonized indicator, and we return to the resulting measurement heterogeneity in the Discussion.

The main adjustment set included age, sex, education, marital status, smoking, drinking, hypertension, diabetes, heart disease, stroke, arthritis, lung disease, cancer, and recent falls (S13 Table in S1 File). These covariates were selected based on a directed acyclic graph (DAG; Fig 1) that encoded the assumed causal structure among the exposure, mediators, outcomes, and confounders. Marital status was included because it proxies social support and socioeconomic resources that influence both oral health-care access (and hence tooth loss) and the maintenance of functional capacity. In the DAG, age, sex, education, and health behaviors were treated as common causes of both edentulism and disability; chronic conditions were modeled as potential confounders; and grip strength, BMI, and depressive symptoms were positioned as mediators on the pathway from edentulism to disability. We acknowledge that grip strength could also act as a confounder if low grip strength preceded tooth loss; however, in the cross-sectional setting, we treated it as a mediator based on the predominant temporal ordering suggested by the literature [14,15], and we report a lagged sensitivity analysis (S12 Table in S1 File) that examines this assumption directly.

**Fig 1:**
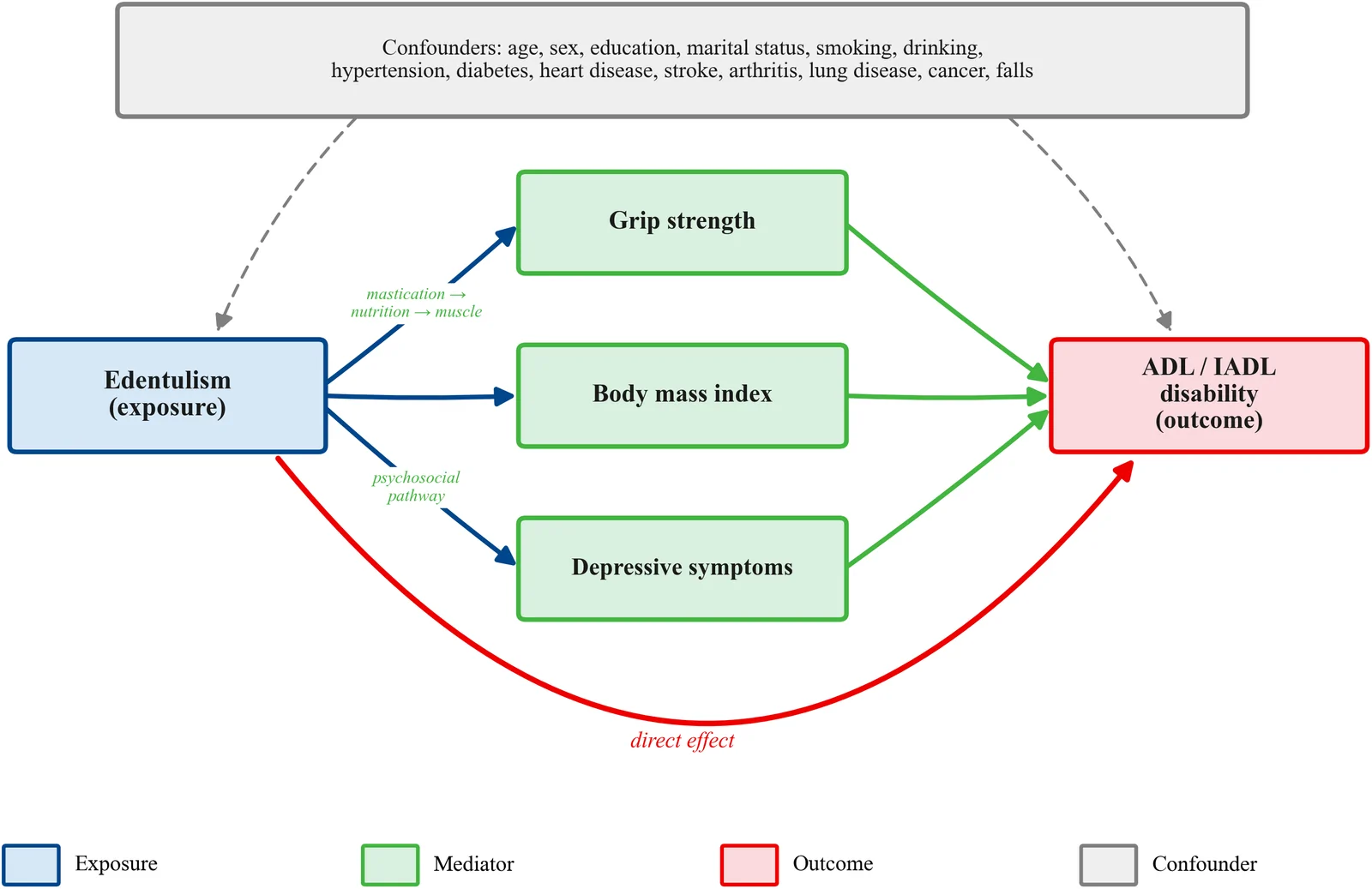
Directed acyclic graph of the hypothesized relationships between edentulism and functional disability.

### Data preprocessing

Each cohort was first harmonized to a common variable structure. Missing values were then handled in the cross-sectional analytic snapshots using an iterative imputer with random-forest regression as a missForest-style proxy [27]. Variables with more than 90% missingness were excluded from imputation, whereas core exposure, outcome, mediator, and covariate fields were retained whenever feasible. This procedure yielded a single imputed analytic dataset for each cohort. Continuous variables were kept on their original scales, and binary or ordinal variables were rounded back to admissible values after imputation. Potential outliers were flagged with an interquartile-range rule and retained in the main analyses.

### Propensity score estimation and diagnostics

Propensity scores for edentulism were estimated with logistic regression including all covariates listed above. No interaction or nonlinear terms were included. Model discrimination was assessed with the C-statistic (area under the receiver operating characteristic curve). Stabilized inverse probability weights were calculated with trimming at the 1st and 99th percentiles of the propensity score distribution. Weight diagnostics included the distribution of stabilized weights (mean, median, range, and percentiles) and the percentage of extreme weights exceeding 10. Covariate balance before and after weighting was evaluated using the absolute standardized mean difference (SMD), with a threshold of 0.10 indicating adequate balance [28–31].

The propensity score model achieved C-statistics of 0.76 in ELSA, 0.73 in HRS, and 0.68 in LASI, indicating moderate discrimination appropriate for confounding control. After IPW, all covariate SMDs were reduced to below 0.05 in every cohort (S2 Table and S1 Fig in S1 File). The mean stabilized weight was close to 1.0 in all cohorts (ELSA: 1.00; HRS: 1.00; LASI: 1.00), and extreme weights exceeding 10 were rare (ELSA: 0.02%; HRS: 0%; LASI: 0%) (S11 Table in S1 File).

### Statistical analysis

All analyses were performed in Python version 3.11 (Python Software Foundation) using the statsmodels, scikit-learn, and lifelines packages; random-effects meta-analysis was performed with the metafor package in R version 4.3; causal mediation analysis was performed with the mediation package (version 4.5.1) in R version 4.5.2. The threshold for statistical significance was two-sided α = 0.05. Weighted logistic regression was used to estimate odds ratios for ADL and IADL disability. As a doubly robust check, we also applied augmented inverse probability weighting (AIPW) [32]. In this step, the propensity model and the outcome models were both fit with logistic regression, and the average treatment effect on the risk scale was converted to an odds ratio. Ninety-five percent confidence intervals for AIPW were obtained with 500 nonparametric bootstrap resamples.

Between-cohort synthesis was performed with random-effects meta-analysis (DerSimonian–Laird) for the main IPW estimates and for the longitudinal Cox estimates [33]. Heterogeneity was described with the *I*^2^ statistic and its corresponding heterogeneity test.

### Longitudinal analysis and Cox model assumptions

Longitudinal analyses used Cox proportional hazards models in disability-free ELSA and HRS participants. Follow-up time was derived from wave differences and expressed in approximate years by treating one wave interval as two years. The proportional hazards assumption was tested for each covariate using scaled Schoenfeld residuals [34]. For the exposure variable (edentulism), the assumption was satisfied in all four models (ELSA ADL: P = 0.98; ELSA IADL: P = 0.46; HRS ADL: P = 0.79; HRS IADL: P = 0.64). Some time-varying effects were observed for age and arthritis, which is expected given the long follow-up; however, because the primary exposure satisfied the assumption and the overall model fit was adequate, no time-dependent extensions were applied.

### Mediation analysis

Mediation was evaluated separately for grip strength, BMI, and depressive symptoms using a counterfactual (potential-outcomes) framework [35–37]. For each mediator we fit a mediator model (linear regression for the continuous mediators grip strength and BMI; logistic regression for the binary mediator depressive symptoms) and an outcome model (logistic regression for the binary disability outcomes), and estimated the average causal mediation effect (ACME, the natural indirect effect, NIE), the average direct effect (ADE, the natural direct effect, NDE), and the total effect (TE) on the risk-difference (probability) scale by counterfactual prediction (g-computation), with 1,000 quasi-Bayesian simulations yielding 95% percentile intervals. Because effects are obtained by counterfactual prediction rather than as a product of regression coefficients, the decomposition satisfies NIE + NDE = TE even for binary outcomes, avoiding the non-collapsibility of the odds-ratio scale that invalidates the product-of-coefficients approach for common binary outcomes such as disability [38]. The proportion mediated (PM%) was reported as a secondary, descriptive measure and interpreted with caution when the total effect was small. The three mediators were analyzed in separate models; therefore, the mediated proportions should not be summed across pathways, as shared variance between mediators would lead to double counting. A lagged sensitivity analysis (exposure, mediator, and outcome measured in separate waves) is reported in S12 Table in S1 File.

### Sensitivity analyses

Several sensitivity analyses were prespecified. First, we repeated the cross-sectional IPW analysis using an alternative snapshot strategy based on the first eligible observation and a second strategy combining the last outcome snapshot with baseline exposure status. Second, because person-level survey weights were available in the HRS analytic file, we reran the HRS IPW analysis using those weights. Third, where observed denture data were available, we compared edentulous participants with and without dentures against non-edentulous participants and then compared denture use versus no denture use within the edentulous group. Fourth, to assess the sensitivity of results to the imputation strategy, we conducted a multiple imputation analysis using five imputed datasets with Rubin’s rules for pooling point estimates and variance components [39,40]. This was compared with the primary single-imputation analysis and a complete-case analysis. We also reviewed E-value and subgroup outputs as supportive robustness checks [41] (S3 Table and S4 Table in S1 File).

As a secondary, supportive analysis, we compared five prediction models for each outcome in each cohort: logistic regression, random forest, ExtraTrees, XGBoost, and LightGBM. Performance was summarized with the area under the receiver operating characteristic curve, Brier score, and average precision. SHAP rankings were used only to check whether the leading predictive features were broadly aligned with the mechanistic findings from the main analyses [42]. Full machine learning results are reported in the supporting information (S8 Table, S9 Table, S2 Fig, S3 Fig in S1 File).

### Ethics statement

This study was a secondary analysis of de-identified cohort data. ELSA received ethical approval from the South Central–Berkshire Research Ethics Committee and from the London Multi-Centre Research Ethics Committee in earlier waves [20]. HRS was approved by the University of Michigan Institutional Review Board [21]. LASI received ethical approvals from the Indian Council of Medical Research (ICMR), the International Institute for Population Sciences (IIPS), the Harvard T. H. Chan School of Public Health, and the University of Southern California [22]. All participants in the parent studies provided written informed consent. Because the present work used publicly available, de-identified secondary data, no additional ethical approval was required for this analysis. The harmonized ELSA, HRS, and LASI datasets used in this study were accessed for research purposes on 12 January 2026. The authors had no access to any information that could identify individual participants at any point during or after data access; all three cohort datasets were released to the authors in fully de-identified form through their respective data custodians (UK Data Service for ELSA, the Health and Retirement Study data distribution at the University of Michigan for HRS, and the International Institute for Population Sciences for LASI).

## Results

### Sample characteristics

The final cross-sectional analytic sample included 63,074 older adults, of whom 1,824 in ELSA, 5,557 in HRS, and 3,562 in LASI were edentulous (Fig 2). This corresponds to edentulism prevalences of 20.0%, 25.1%, and 11.2%, respectively. The longitudinal samples contained 6,384 disability-free ELSA participants and 16,284 disability-free HRS participants before the complete-case requirements of the Cox models were applied (5,323 and 13,131 after exclusions).

**Fig 2:**
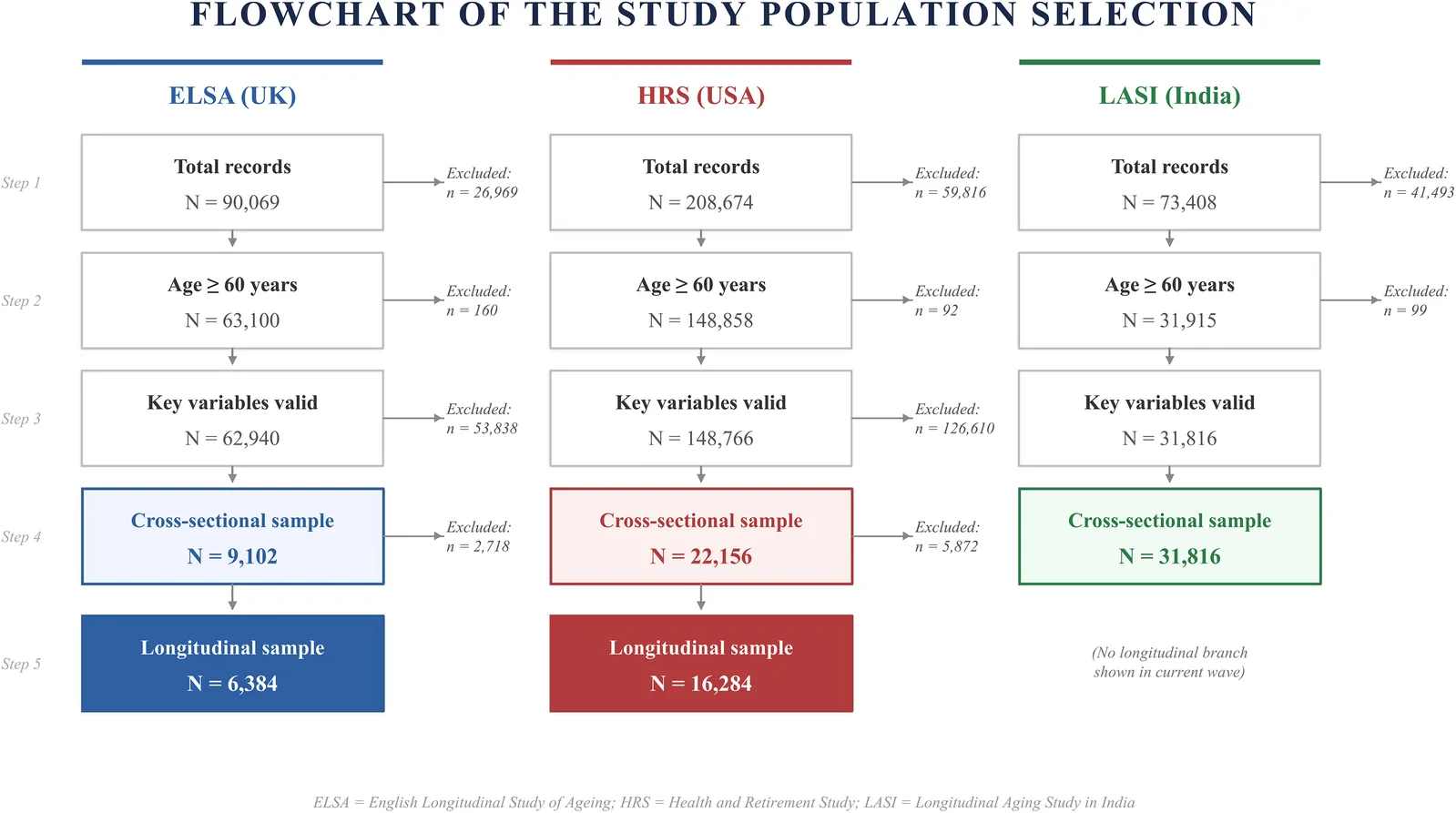
Study flow chart for the cross-sectional and longitudinal analytic samples.

Across all three cohorts, the edentulous group was older and had lower grip strength than the non-edentulous group. In ELSA, the median age was 81.0 years in the edentulous group versus 75.0 years in the non-edentulous group, and median grip strength was 20.0 kg versus 24.1 kg. Similar gradients were seen in HRS and LASI. Depressive symptom burden was also generally higher among edentulous participants, while smoking and multimorbidity were more common in several cohorts (Table 1).

**Table 1:** Baseline characteristics by edentulism status in each cohort.

Cohort	Variable	Edentulous	Non-edentulous	P value	SMD
ELSA	N	1,824	7,278		
ELSA	Age (years)	81.0 [74.0, 87.0]	75.0 [70.0, 81.0]	<0.001	0.609
ELSA	Grip strength (kg)	20.0 [14.0, 25.3]	24.1 [19.8, 33.2]	<0.001	−0.603
ELSA	BMI (kg/m²)	27.0 [25.1, 28.7]	27.4 [26.2, 28.8]	<0.001	−0.170
ELSA	CES-D score	1.4 [0.0, 3.0]	1.0 [0.0, 2.0]	<0.001	0.264
ELSA	Male sex	724 (39.7%)	3,485 (47.9%)	<0.001	−0.166
ELSA	Married/partnered	768 (42.1%)	4,501 (61.8%)	<0.001	−0.403
ELSA	Current smoker	269 (14.7%)	537 (7.4%)	<0.001	0.237
ELSA	Current drinker	1,236 (67.8%)	6,064 (83.3%)	<0.001	−0.368
ELSA	Hypertension	1,108 (60.7%)	3,962 (54.4%)	<0.001	0.128
ELSA	Diabetes	368 (20.2%)	1,111 (15.3%)	<0.001	0.129
ELSA	Heart disease	704 (38.6%)	2,371 (32.6%)	<0.001	0.126
ELSA	Stroke	292 (16.0%)	636 (8.7%)	<0.001	0.222
ELSA	Arthritis	1,044 (57.2%)	3,552 (48.8%)	<0.001	0.170
ELSA	Lung disease	308 (16.9%)	677 (9.3%)	<0.001	0.226
ELSA	Cancer	315 (17.3%)	1,304 (17.9%)	0.540	−0.017
ELSA	Fall history	755 (41.4%)	2,343 (32.2%)	<0.001	0.192
ELSA	Depression	382 (20.9%)	977 (13.4%)	<0.001	0.200
ELSA	ADL disability (≥1)	776 (42.5%)	1,874 (25.7%)	<0.001	0.360
ELSA	IADL disability (≥1)	778 (42.7%)	1,500 (20.6%)	<0.001	0.488
ELSA	Education: less than high school	1,238 (67.9%)	2,493 (34.3%)	<0.001	
ELSA	Education: high school	516 (28.3%)	3,652 (50.2%)		
ELSA	Education: college or higher	70 (3.8%)	1,133 (15.6%)		
HRS	N	5,557	16,599		
HRS	Age (years)	78.0 [69.0, 85.0]	75.0 [68.0, 83.0]	<0.001	0.189
HRS	Grip strength (kg)	22.1 [17.8, 30.0]	25.0 [19.9, 32.6]	<0.001	−0.275
HRS	BMI (kg/m^2^)	28.3 [26.1, 30.5]	28.6 [26.6, 30.4]	<0.001	−0.050
HRS	CES-D score	1.3 [0.7, 3.0]	1.0 [0.0, 2.0]	<0.001	0.260
HRS	Male sex	2,343 (42.2%)	7,146 (43.1%)	0.253	−0.018
HRS	Married/partnered	2,111 (38.0%)	8,573 (51.6%)	<0.001	−0.277
HRS	Current smoker	944 (17.0%)	1,253 (7.5%)	<0.001	0.291
HRS	Current drinker	1,789 (32.2%)	8,269 (49.8%)	<0.001	−0.364
HRS	Hypertension	4,281 (77.0%)	11,600 (69.9%)	<0.001	0.163
HRS	Diabetes	1,996 (35.9%)	5,032 (30.3%)	<0.001	0.119
HRS	Heart disease	2,510 (45.2%)	5,930 (35.7%)	<0.001	0.193
HRS	Stroke	1,246 (22.4%)	2,423 (14.6%)	<0.001	0.202
HRS	Arthritis	4,219 (75.9%)	11,486 (69.2%)	<0.001	0.151
HRS	Lung disease	1,434 (25.8%)	2,168 (13.1%)	<0.001	0.326
HRS	Cancer	1,307 (23.5%)	3,823 (23.0%)	0.466	0.012
HRS	Fall history	2,543 (45.8%)	6,351 (38.3%)	<0.001	0.152
HRS	Depression	1,097 (19.7%)	2,313 (13.9%)	<0.001	0.156
HRS	ADL disability (≥1)	2,359 (42.5%)	4,560 (27.5%)	<0.001	0.318
HRS	IADL disability (≥1)	2,475 (44.5%)	4,539 (27.3%)	<0.001	0.364
HRS	Education: less than high school	2,165 (39.0%)	2,831 (17.1%)	<0.001	
HRS	Education: high school	3,022 (54.4%)	9,588 (57.8%)		
HRS	Education: college or higher	370 (6.7%)	4,180 (25.2%)		
LASI	N	3,562	28,254		
LASI	Age (years)	72.0 [66.0, 79.0]	67.0 [63.0, 72.0]	<0.001	0.599
LASI	Grip strength (kg)	18.0 [14.0, 23.5]	20.5 [16.0, 26.0]	<0.001	−0.312
LASI	BMI (kg/m²)	20.8 [18.4, 23.9]	21.7 [19.0, 24.8]	<0.001	−0.151
LASI	CES-D score	3.0 [2.0, 4.0]	3.0 [2.0, 4.0]	0.157	0.044
LASI	Male sex	1,566 (44.0%)	13,718 (48.6%)	<0.001	−0.092
LASI	Married/partnered	1,842 (51.7%)	18,481 (65.4%)	<0.001	−0.281
LASI	Current smoker	597 (16.8%)	3,800 (13.4%)	<0.001	0.093
LASI	Current drinker	529 (14.9%)	4,897 (17.3%)	<0.001	−0.068
LASI	Hypertension	1,293 (36.3%)	9,927 (35.1%)	0.176	0.024
LASI	Diabetes	542 (15.2%)	4,389 (15.5%)	0.639	−0.009
LASI	Heart disease	212 (6.0%)	1,383 (4.9%)	0.007	0.047
LASI	Stroke	108 (3.0%)	735 (2.6%)	0.146	0.026
LASI	Arthritis	471 (13.2%)	3,976 (14.1%)	0.176	−0.025
LASI	Lung disease	110 (3.1%)	728 (2.6%)	0.082	0.031
LASI	Cancer	33 (0.9%)	204 (0.7%)	0.217	0.023
LASI	Fall history	829 (23.3%)	5,552 (19.7%)	<0.001	0.088
LASI	Depression	1,026 (28.8%)	7,583 (26.8%)	0.014	0.044
LASI	ADL disability (≥1)	1,110 (31.2%)	5,526 (19.6%)	<0.001	0.269
LASI	IADL disability (≥1)	1,777 (49.9%)	10,782 (38.2%)	<0.001	0.238
LASI	Education: less than high school	2,905 (81.6%)	21,879 (77.4%)	<0.001	
LASI	Education: high school	552 (15.5%)	5,170 (18.3%)		
LASI	Education: college or higher	105 (2.9%)	1,205 (4.3%)		

Values are median [interquartile range] or count (%). *P* values compare edentulous and non-edentulous participants within each cohort. BMI, body mass index; CES-D, Center for Epidemiologic Studies Depression Scale; SMD, standardized mean difference.

Disability prevalence was consistently higher among participants without teeth. ADL disability affected 42.5% of edentulous participants in both ELSA and HRS and 31.2% in LASI; the corresponding prevalences among non-edentulous participants were 25.7%, 27.5%, and 19.6%. For IADL disability, the prevalences were 42.7% versus 20.6% in ELSA, 44.5% versus 27.3% in HRS, and 49.9% versus 38.2% in LASI.

### Cross-sectional associations

In the primary IPW analysis, edentulism was associated with higher odds of ADL disability in all three cohorts: 1.30 (95% confidence interval 1.17–1.46) in ELSA, 1.23 (1.15–1.31) in HRS, and 1.40 (1.29–1.51) in LASI. The pooled cross-national estimate was 1.30 (1.20–1.42), with moderate heterogeneity (I2 = 65.3%, P = 0.06). The pattern was similar for IADL disability, with cohort-specific odds ratios of 1.41 (1.26–1.58), 1.31 (1.23–1.40), and 1.12 (1.04–1.20), respectively. The pooled IADL estimate was 1.27 (1.11–1.45), although heterogeneity was higher (I2 = 87.8%, P < 0.001), largely because the LASI effect was weaker than those in ELSA and HRS (Table 2).

**Table 2:** Main cross-sectional and longitudinal estimated associations for edentulism and functional disability.

Outcome	Cohort	Method	Effect	Estimate (95% CI)	P value	N	I²
ADL	ELSA	IPW	OR	1.30 (1.17, 1.46)	<0.001	9,102	
ADL	ELSA	AIPW	OR	1.28 (1.14, 1.45)	<0.001	9,102	
ADL	HRS	IPW	OR	1.23 (1.15, 1.31)	<0.001	22,156	
ADL	HRS	AIPW	OR	1.21 (1.14, 1.29)	<0.001	22,156	
ADL	LASI	IPW	OR	1.40 (1.29, 1.51)	<0.001	31,816	
ADL	LASI	AIPW	OR	1.38 (1.28, 1.49)	<0.001	31,816	
ADL	Pooled	IPW meta	OR	1.30 (1.20, 1.42)		63,074	65.3%
IADL	ELSA	IPW	OR	1.41 (1.26, 1.58)	<0.001	9,102	
IADL	ELSA	AIPW	OR	1.38 (1.24, 1.56)	<0.001	9,102	
IADL	HRS	IPW	OR	1.31 (1.23, 1.40)	<0.001	22,156	
IADL	HRS	AIPW	OR	1.29 (1.20, 1.37)	<0.001	22,156	
IADL	LASI	IPW	OR	1.12 (1.04, 1.20)	0.003	31,816	
IADL	LASI	AIPW	OR	1.10 (1.03, 1.18)	0.011	31,816	
IADL	Pooled	IPW meta	OR	1.27 (1.11, 1.45)		63,074	87.8%
ADL	ELSA	Cox	HR	1.11 (0.98, 1.27)	0.110	5,323	
ADL	HRS	Cox	HR	1.23 (1.14, 1.32)	<0.001	13,131	
ADL	Pooled	Cox meta	HR	1.19 (1.09, 1.30)		18,454	35.7%
IADL	ELSA	Cox	HR	1.22 (1.05, 1.41)	0.009	5,323	
IADL	HRS	Cox	HR	1.34 (1.25, 1.44)	<0.001	13,131	
IADL	Pooled	Cox meta	HR	1.30 (1.19, 1.43)		18,454	30.6%

AIPW confidence intervals were obtained with 500 bootstrap resamples. Cox models were fit only in ELSA and HRS participants who were free of both ADL and IADL disability at baseline. *I*^2^ is shown for pooled estimates only. AIPW, augmented inverse probability weighting; CI, confidence interval; Cox, Cox proportional hazards; HR, hazard ratio; IPW, inverse probability weighting; OR, odds ratio.

AIPW estimates supported the same direction of effect in every cohort. For ADL disability, the AIPW odds ratios were 1.28 (1.14–1.45) in ELSA, 1.21 (1.14–1.29) in HRS, and 1.38 (1.28–1.49) in LASI. For IADL disability, the corresponding estimates were 1.38 (1.24–1.56), 1.29 (1.20–1.37), and 1.10 (1.03–1.18). The similarity between the IPW and AIPW findings suggests that the main conclusion was not sensitive to the choice between weighting alone and a doubly robust estimator (Fig 3).

**Fig 3:**
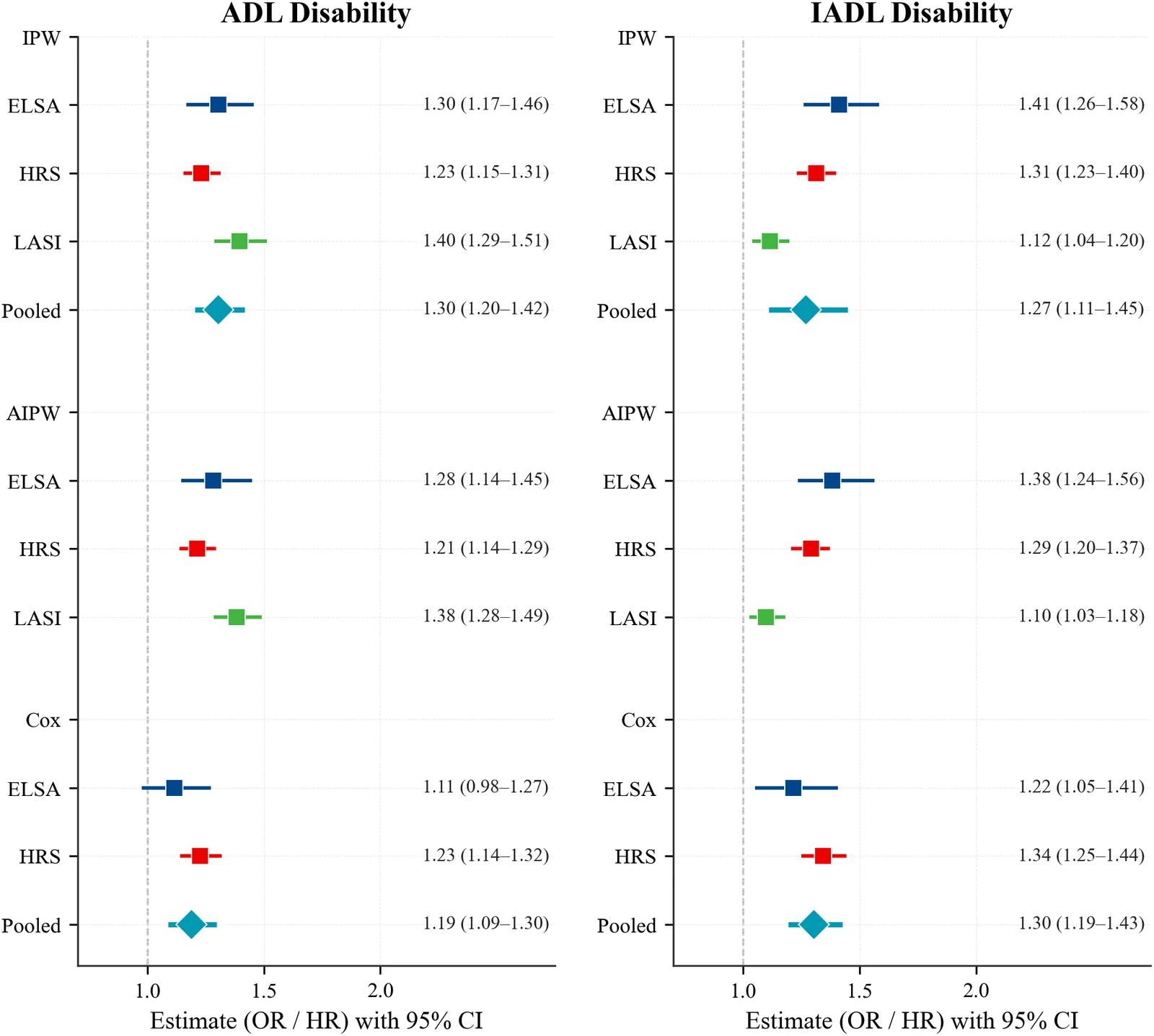
Cross-national effect estimates for edentulism and functional disability from IPW, AIPW, and Cox models.

### Longitudinal associations

Longitudinal analyses pointed in the same direction. In disability-free participants, edentulism predicted incident ADL disability with a pooled hazard ratio of 1.19 (1.09–1.30; *I*^2^ = 35.7%, *P* = 0.21). The cohort-specific hazard ratios were 1.11 (0.98–1.27) in ELSA and 1.23 (1.14–1.32) in HRS. The ELSA estimate did not reach conventional statistical significance (*P* = 0.11), which may reflect the smaller longitudinal sample size (*n* = 5,323) and longer follow-up with competing risks. For incident IADL disability, the pooled hazard ratio was 1.30 (1.19–1.43; *I*^2^ = 30.6%, *P* = 0.23), with cohort-specific estimates of 1.22 (1.05–1.41) and 1.34 (1.25–1.44), respectively. Median follow-up was 8 years in ELSA and 6–8 years in HRS, depending on the outcome. The proportional hazards assumption was satisfied for the edentulism variable in all four Cox models (Schoenfeld test P > 0.46 in all cases; S10 Table in S1 File). Kaplan–Meier curves illustrating the divergence in disability-free survival are shown in Fig 4.

**Fig 4:**
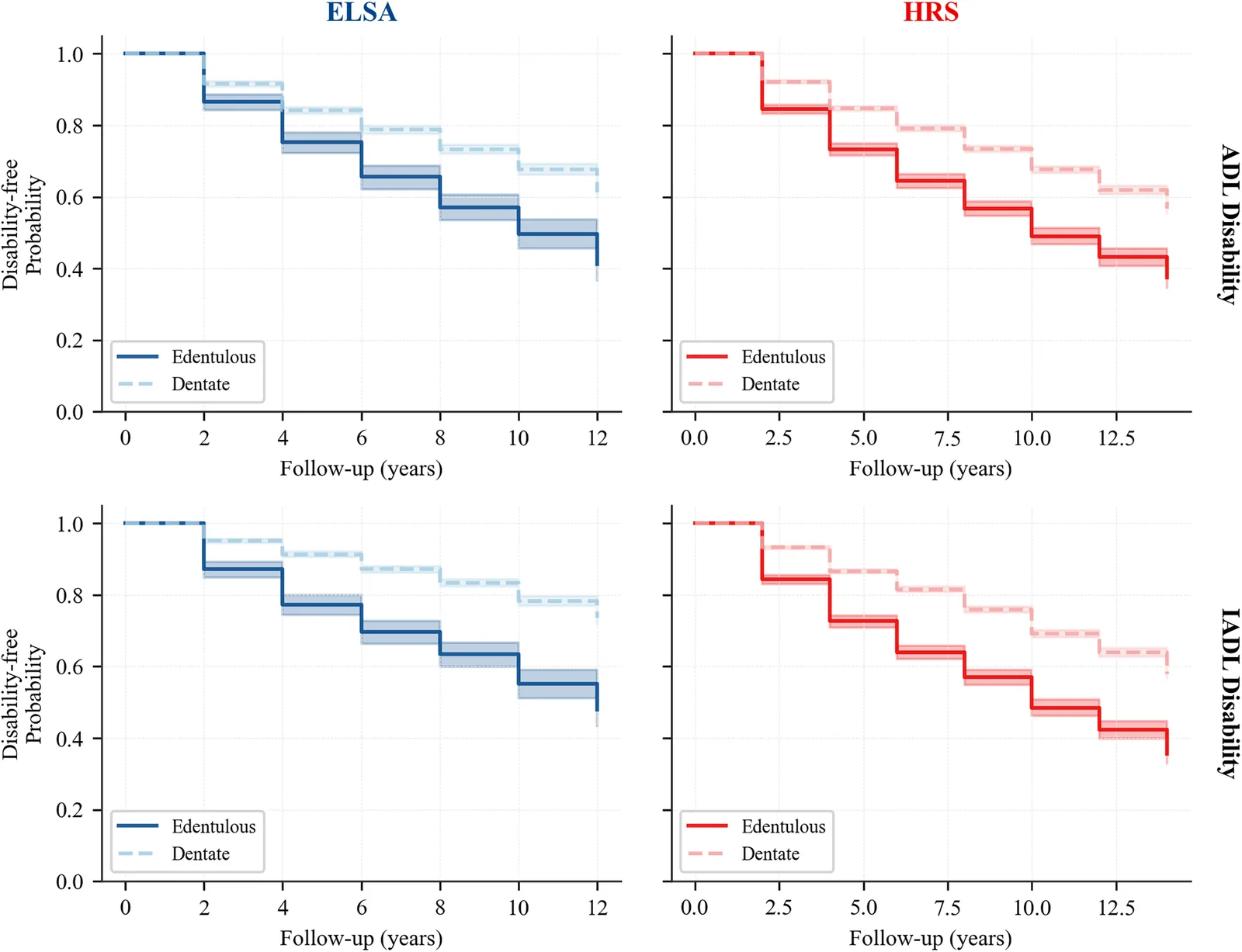
Kaplan–Meier survival curves for incident ADL and IADL disability by edentulism status in ELSA and HRS.

### Mediation analyses

Grip strength showed the largest proportion mediated across settings. For ADL disability, the proportion mediated by grip strength was 52.8% (95% confidence interval 32.4–113.2) in ELSA, 52.0% (37.8–80.2) in HRS, and 5.4% (2.5–9.4) in LASI. For IADL disability, the corresponding estimates were 44.9% (30.2–71.6), 45.4% (34.8–61.5), and 13.3% (5.9–32.5). Depressive symptoms mediated a small and mostly non-significant share (e.g., 6.0% in ELSA ADL), and BMI mediated close to none. Lower muscle strength thus accounted for a substantial share of the association in the English and US cohorts and a smaller share in LASI (Table 3).

**Table 3:** Mediation results for grip strength, BMI, and depressive symptoms.

Outcome	Cohort	Pathway	NIE (95% CI)	NDE (95% CI)	TE	PM (%)	N
ADL	ELSA	Grip	0.021 (0.013, 0.028)	0.018 (−0.003, 0.039)	0.039	52.8 (32.4, 113.2)	9,102
ADL	ELSA	BMI	0.000 (−0.001, 0.002)	0.039 (0.016, 0.061)	0.039	0.1 (−4.8, 4.2)	9,102
ADL	ELSA	Depression	0.002 (−0.000, 0.005)	0.037 (0.014, 0.059)	0.039	6.0 (−1.4, 17.0)	9,102
ADL	HRS	Grip	0.018 (0.014, 0.022)	0.017 (0.005, 0.028)	0.035	52.0 (37.8, 80.2)	22,156
ADL	HRS	BMI	0.000 (−0.002, 0.002)	0.035 (0.022, 0.048)	0.035	0.1 (−7.7, 6.7)	22,156
ADL	HRS	Depression	0.002 (−0.000, 0.004)	0.034 (0.021, 0.046)	0.035	4.9 (−0.6, 11.3)	22,156
ADL	LASI	Grip	0.003 (0.001, 0.005)	0.052 (0.038, 0.066)	0.055	5.4 (2.5, 9.4)	31,816
ADL	LASI	BMI	0.000 (−0.000, 0.001)	0.055 (0.041, 0.070)	0.056	0.3 (−0.1, 0.9)	31,816
ADL	LASI	Depression	0.000 (−0.002, 0.002)	0.055 (0.041, 0.070)	0.056	0.4 (−2.9, 3.4)	31,816
IADL	ELSA	Grip	0.022 (0.015, 0.030)	0.027 (0.009, 0.045)	0.050	44.9 (30.2, 71.6)	9,102
IADL	ELSA	BMI	−0.000 (−0.001, 0.001)	0.054 (0.033, 0.074)	0.054	−0.1 (−2.2, 2.1)	9,102
IADL	ELSA	Depression	0.003 (−0.001, 0.006)	0.051 (0.031, 0.071)	0.054	4.9 (−1.1, 11.2)	9,102
IADL	HRS	Grip	0.021 (0.017, 0.026)	0.025 (0.014, 0.036)	0.047	45.4 (34.8, 61.5)	22,156
IADL	HRS	BMI	0.000 (−0.001, 0.001)	0.048 (0.035, 0.061)	0.048	0.0 (−2.2, 2.1)	22,156
IADL	HRS	Depression	0.001 (−0.000, 0.003)	0.047 (0.034, 0.059)	0.048	2.9 (−0.3, 6.6)	22,156
IADL	LASI	Grip	0.004 (0.002, 0.007)	0.027 (0.011, 0.044)	0.032	13.3 (5.9, 32.5)	31,816
IADL	LASI	BMI	0.001 (0.000, 0.002)	0.031 (0.014, 0.047)	0.032	3.9 (0.8, 10.6)	31,816
IADL	LASI	Depression	0.000 (−0.002, 0.002)	0.032 (0.015, 0.049)	0.032	0.7 (−7.2, 8.0)	31,816

Effects are average causal mediation effects on the risk-difference (probability) scale from counterfactual mediation analysis (R mediation package, 1,000 quasi-Bayesian simulations). NIE, natural indirect effect (average causal mediation effect); NDE, natural direct effect (average direct effect); TE, total effect; PM, proportion mediated. Because the total effect is small for some cohort-outcome combinations, PM can be unstable and may occasionally yield confidence bounds exceeding 100%; such estimates should be interpreted as indicating the direction and approximate magnitude of mediation.

Depressive symptoms showed little mediation overall. In the counterfactual analysis the depression-mediated proportion was small and predominantly non-significant: 6.0% (−1.4 to 17.0) in ELSA and 4.9% (−0.6 to 11.3) in HRS for ADL disability, and 4.9% (−1.1 to 11.2) and 2.9% (−0.3 to 6.6) for IADL disability, with confidence intervals crossing the null in most cohort-outcome combinations. In LASI, the depression-mediated proportion was close to zero (ADL 0.4%, −2.9 to 3.4; IADL 0.7%, −7.2 to 8.0). BMI contributed little in all cohorts; only the LASI IADL model showed a small mediated proportion of 3.9% (0.8–10.6).

The mediated proportions for grip strength and depressive symptoms were estimated in separate counterfactual models and should not be summed. For example, in ELSA the grip-mediated proportion for ADL disability was 52.8% and the depression-mediated proportion was 6.0%, reflecting distinct pathways rather than a combined total. Because the total effect is small for some cohort-outcome combinations, proportion-mediated estimates can be unstable, occasionally with upper confidence bounds exceeding 100%; these are reported on the risk-difference scale under counterfactual decomposition and should be interpreted as indicating the direction and approximate magnitude of mediation rather than precise causal partitioning.

The mediation results suggest that the pathway from edentulism to disability is more plausibly carried through functional reserve, particularly muscle strength, than through body size or depressive symptoms. The muscular pathway accounted for the largest share of the association, whereas BMI and depressive symptoms showed little mediation (Fig 5).

**Fig 5:**
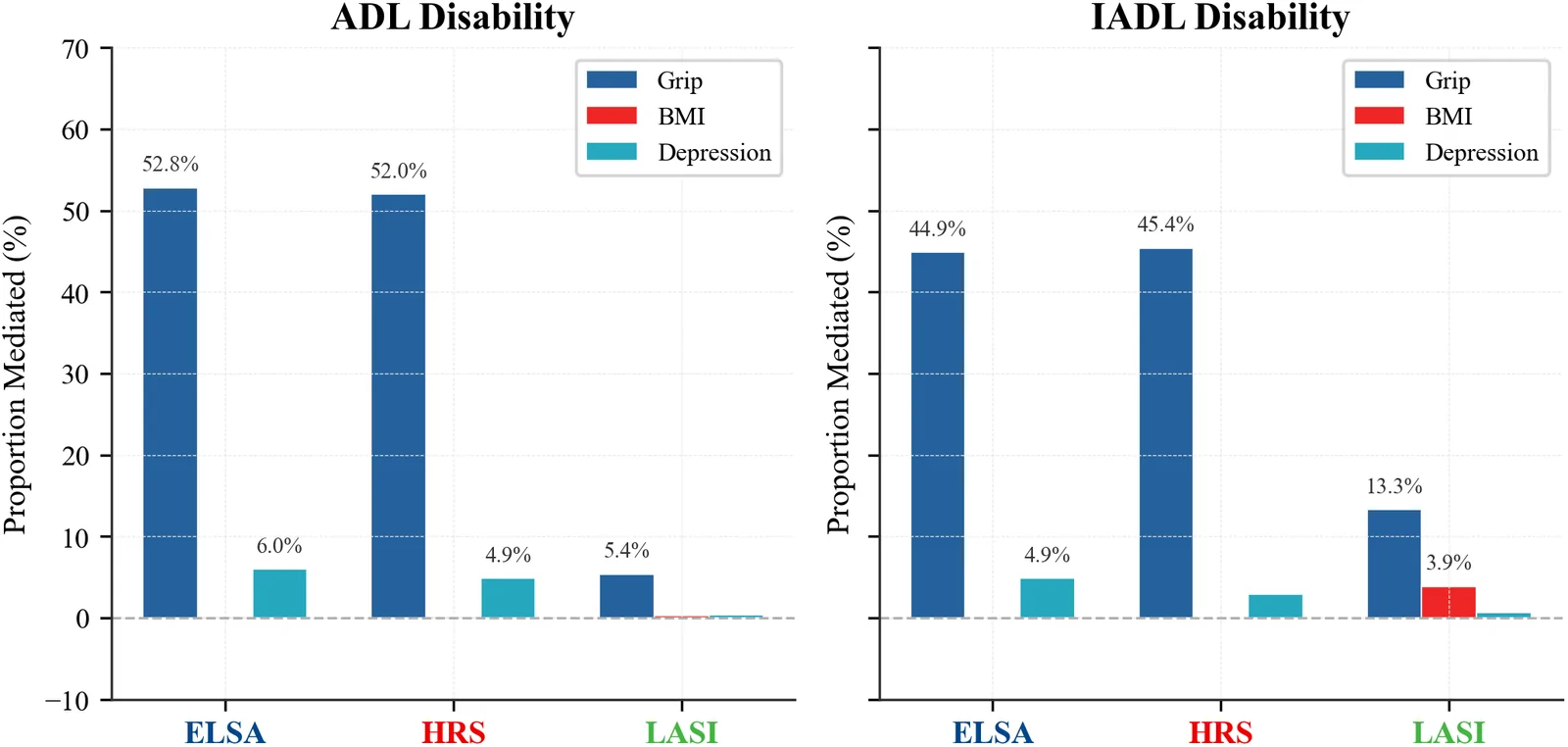
Summary of mediation proportions for grip strength, BMI, and depressive symptoms across cohorts.

### Sensitivity and supportive analyses

The main findings proved stable across multiple sensitivity checks. In the multiple imputation analysis (five imputed datasets, Rubin’s rules), the pooled IPW odds ratios were virtually identical to the primary single-imputation estimates. For ADL disability, the multiple imputation ORs were 1.31 (1.17–1.46) in ELSA, 1.23 (1.16–1.32) in HRS, and 1.40 (1.29–1.51) in LASI; the corresponding single-imputation ORs were 1.30 (1.17–1.46), 1.23 (1.15–1.31), and 1.40 (1.29–1.51). For IADL disability, the agreement was equally close: multiple imputation yielded 1.41 (1.26–1.58) in ELSA, 1.32 (1.23–1.40) in HRS, and 1.11 (1.04–1.20) in LASI. Complete-case analyses also produced consistent results (ELSA ADL: 1.38; HRS: 1.23; LASI: 1.40), with slightly wider confidence intervals reflecting the reduced sample sizes (S1 Table in S1 File).

When the cross-sectional snapshot was redefined, the odds ratios remained elevated. Using the first eligible snapshot, the ADL odds ratios were 1.21 in ELSA, 1.15 in HRS, and 1.40 in LASI. Using baseline exposure with the last outcome snapshot produced nearly identical estimates to the main analysis. IADL findings changed little under either alternative definition (S5 Table in S1 File).

The survey-weighted HRS analysis also supported the main result. The weighted odds ratio was 1.31 (1.21–1.42) for ADL disability and 1.44 (1.33–1.56) for IADL disability, indicating that the association was not explained by ignoring the complex sampling structure of HRS in the main model (S6 Table in S1 File).

Observed denture information was available at usable frequency only in LASI. Compared with non-edentulous participants, edentulous LASI participants without dentures had higher odds of ADL disability (1.52, 1.39–1.66) and IADL disability (1.26, 1.16–1.37). By contrast, edentulous participants who reported dentures showed little excess ADL risk (1.02, 0.85–1.23) and a lower IADL estimate than the untreated edentulous group (0.83, 0.70–0.98 relative to non-edentulous participants). Within the edentulous subset, denture use was associated with lower odds of ADL disability (0.66, 0.54–0.82) and IADL disability (0.65, 0.53–0.78). Because this analysis was cross-sectional, confined to a single cohort, and especially susceptible to confounding by the resources and health-seeking behaviour that determine who receives dentures, it should be regarded as an exploratory, hypothesis-generating finding rather than as definitive (Fig 6; S7 Table in S1 File).

**Fig 6:**
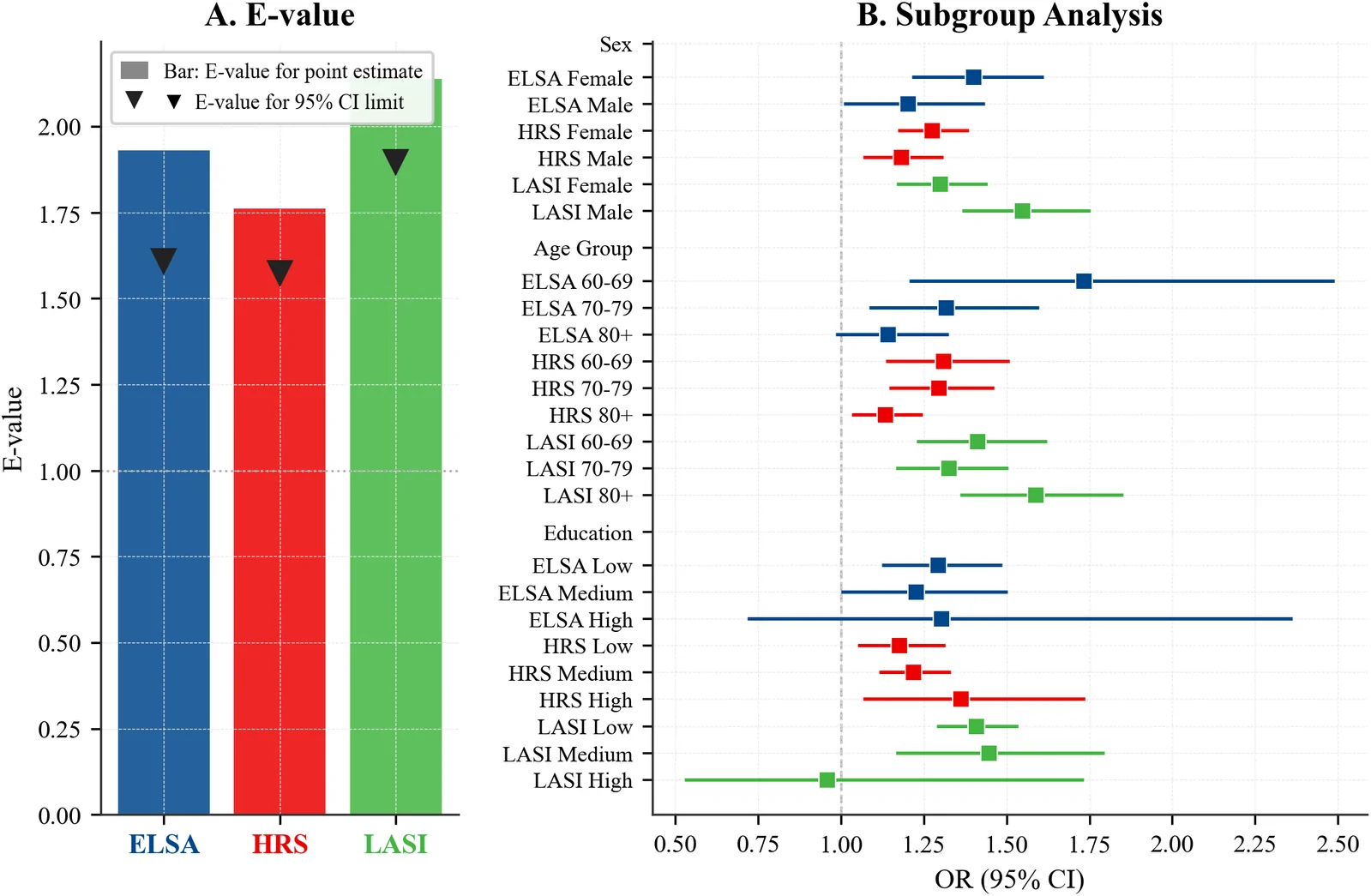
Sensitivity analyses for alternative wave definitions, survey weighting, and denture status.

The supportive prediction analysis showed internal consistency with the main findings. LightGBM produced the best discrimination in all six cohort–outcome combinations, with area under the curve values ranging from 0.724 to 0.917 for ADL disability and from 0.734 to 0.927 for IADL disability. SHAP rankings repeatedly placed grip strength, depressive symptoms, and age near the top, which aligned with the mechanism-focused findings from the main analysis (S8 Table, S9 Table, S2 Fig, S3 Fig in S1 File).

## Discussion

This analysis yields three main points. First, edentulism was associated with higher odds of both ADL and IADL disability in England, the United States, and India. The signal was visible in every cohort and persisted when we changed the estimator, switched to a longitudinal design where possible, redefined the cross-sectional snapshot, varied the imputation strategy, and adjusted for marital status. Second, grip strength showed the largest and most consistent mediation proportion across cohorts, although temporality could not be established in the cross-sectional design. Third, the denture results in LASI suggest that oral rehabilitation may matter, although that finding remains exploratory.

The overall direction of the findings is consistent with earlier work while extending it in two ways. Matsuyama and colleagues used a natural experiment framework in ELSA and reported that tooth loss reduced functional capacity in older adults [7]. The JAGES cohort similarly found that poorer oral status predicted incident functional disability [8]. The recent meta-analysis by Ko and colleagues synthesized broader evidence for oral health and oral function as risk factors for disability and mortality [10]. Our study adds to that literature by applying a common analytic framework across three very different ageing cohorts at the individual-participant level and by showing that the pattern survives both cross-sectional weighting and longitudinal follow-up in two countries. This individual-level cross-national harmonization approach offers advantages over aggregate-level meta-analysis because it allows standardized covariate adjustment and direct comparability of effect estimates [23,24].

The mediation results sharpen the biological interpretation. Grip strength accounted for the largest proportion mediated across outcomes, which is compatible with the idea that edentulism contributes to disability partly through impaired mastication, reduced dietary adequacy, and loss of physical reserve. That interpretation fits earlier reports linking lower occlusal force to incident disability and adverse outcomes [11,12] and is consistent with the growing body of evidence connecting oral health to sarcopenia and muscle function [14–16,43–45]. The prominence of grip strength is biologically plausible: chewing efficiency falls sharply after complete tooth loss, which narrows dietary variety and lowers the intake of protein and other nutrients needed to maintain skeletal muscle; the resulting decline in muscle mass and strength then translates into difficulty with the transfers, lifting, and mobility tasks that define ADL and IADL limitation. We therefore interpret grip strength as lying on a plausible mechanistic pathway, although the cross-sectional primary analysis cannot establish temporality. A lagged ELSA/HRS sensitivity analysis with exposure, mediator, and outcome measured 6–8 years apart supported the direction of effect but yielded attenuated mediated proportions, indicating that the cross-sectional estimates may also reflect concurrent decline rather than a purely sequential mechanism. Accordingly, the mediated proportions should be read as indicating the relative importance of the muscular pathway rather than as exact causal quantification. BMI explained little in most models; it is an accessible summary measure but a blunt one that does not distinguish muscle from fat mass and is poorly suited to capture changes in food texture, protein intake, or sarcopenic processes that may follow severe tooth loss [17].

Depressive symptoms showed little mediation in the counterfactual analysis across all cohorts, and the small, predominantly non-significant estimates should be interpreted with caution. The instruments themselves were not identical (ELSA and HRS used an eight-item CES-D symptom count and LASI a ten-item count), and this measurement heterogeneity may limit the comparability of the depression pathway across cohorts. The disability pathway in LASI may also be more strongly shaped by physical workload, socioeconomic adversity, or health-care access than by the psychosocial consequences of tooth loss alone. The depression-mediated proportions should therefore not be over-interpreted.

The difference in grip strength mediation between ELSA/HRS (about 45–53%) and LASI (5–14%) deserves closer examination. Several factors may contribute. First, the LASI population is younger on average and has different baseline grip strength distributions, which may attenuate the mediating role of muscle function. Second, in LASI, edentulism may operate through pathways not captured in our mediator set, such as nutritional deficiency, chronic infection, or reduced social participation, that are more prominent in low- and middle-income settings [46]. Third, the IADL items in LASI may capture somewhat different functional domains than those in ELSA and HRS, potentially emphasizing social participation over physical capacity. These cross-national differences point to the importance of context-specific interpretation and suggest that a single mechanistic model may not fully explain the edentulism-disability link across diverse populations.

The denture analysis deserves a cautious but serious reading. Among edentulous LASI participants, denture use was associated with substantially lower odds of both ADL and IADL disability. This is directionally consistent with studies linking prosthetic rehabilitation, occlusal force, and preserved function in older adults [12,47–49]. Still, the present estimate is observational, cross-sectional, and vulnerable to residual confounding by socioeconomic resources, health-seeking behavior, and access to dental services. Because of these limitations, we present it strictly as an exploratory, hypothesis-generating finding that requires confirmation in prospective or interventional studies. What it offers is not causal confirmation, but a clinically relevant clue: restoration may be one pathway through which disability risk can be mitigated.

The ELSA longitudinal result for ADL disability (hazard ratio 1.11, *P* = 0.11) did not reach conventional statistical significance. This may reflect the smaller ELSA longitudinal sample (*n* = 5,323), longer follow-up with competing mortality risks [50], or a genuinely weaker association in the English context. The HRS longitudinal estimate, by contrast, was clearly significant (hazard ratio 1.23, *P* < 0.001), and the pooled estimate remained significant (hazard ratio 1.19, 1.09–1.30). The ELSA IADL result was significant (hazard ratio 1.22, *P* = 0.009), suggesting that the temporal association is present but may be outcome-specific in smaller samples.

Several strengths should be noted. The study used large, population-based cohorts from three countries, all analyzed under a harmonized framework. The main result was triangulated across IPW, AIPW, and Cox models rather than resting on a single specification. Propensity score diagnostics confirmed adequate covariate balance after weighting (all SMDs < 0.05), and the proportional hazards assumption was formally tested. Multiple imputation sensitivity analysis confirmed that the primary results were not driven by the single-imputation strategy. Mechanism and robustness were addressed directly instead of being deferred to speculation.

At the same time, the work has important limitations. Although we adjusted for a broad covariate set (now including marital status) and used propensity-score weighting, residual confounding remains the central threat to validity. Several factors that can influence both tooth loss and disability, including socioeconomic position and wealth, lifelong nutritional status, access to and use of dental and general health care, frailty, and cognitive function, were only partially captured by the harmonized variables, and unmeasured or imperfectly measured confounding of this kind could account for part of the observed association. The primary cross-sectional analysis depends on the assumption of no unmeasured confounding, and AIPW cannot rescue violations of exchangeability [32]. Reverse causation is also plausible: established disability, and the frailty, poor nutrition, and reduced self-care that accompany it, may themselves accelerate tooth loss, so the cross-sectional association should be regarded as bidirectional in principle. The longitudinal Cox analyses, which required participants to be free of disability at baseline, provide the strongest available evidence on temporal ordering, but they were limited to two cohorts and remain observational. Although multiple imputation produced nearly identical results, the imputation model itself relies on the missing-at-random assumption. LASI contributed only cross-sectional evidence to the main question, and denture status was usable in only one cohort. The mediation findings call for particular caution: although re-estimated within a counterfactual framework on the risk-difference scale, the mediators were measured contemporaneously with the outcome in the primary analysis, so temporality cannot be established; a lagged ELSA/HRS sensitivity analysis (exposure, mediator, and outcome measured 6–8 years apart) supported the assumed direction of effect but yielded attenuated mediated proportions and could not fully rule out reverse causation. LASI contributed only cross-sectional mediation. Proportion-mediated estimates remain unstable when the total effect is small and should be interpreted as approximate [38]. Finally, the IADL items and the depression instruments, while harmonized, may not capture identical constructs across the three cohorts, which could contribute to the observed heterogeneity.

Despite these limitations, the practical implication is straightforward. Complete tooth loss appears to mark broad vulnerability in later-life functioning, not only oral disadvantage. Screening for edentulism may help identify older adults who need closer evaluation of strength, mood, and daily function [51]. The results also argue against treating oral rehabilitation as a purely cosmetic issue in older populations.

## Conclusions

In three large ageing cohorts from England, the United States, and India, edentulism was consistently associated with a higher burden of functional disability. The association was reproduced in weighted cross-sectional models, doubly robust estimation, and longitudinal follow-up. Grip strength showed the largest mediation proportion, consistent with a plausible mechanistic pathway, although temporality could not be established in the cross-sectional design. BMI had limited explanatory value and depressive symptoms showed little mediation. Multiple imputation and alternative analytic strategies confirmed the robustness of the primary findings. The exploratory denture findings suggest that oral rehabilitation may attenuate disability risk, but that question now needs prospective validation.

## Supporting information

S1 Checklist STROBE statement checklist for reporting of observational studies. (DOCX)

S1 File Supplementary methods, supplementary tables, and supplementary figures. Supplementary methods describe the multiple imputation procedure, propensity score model details, Cox proportional hazards assumption testing, and machine learning models. Supplementary tables include multiple imputation sensitivity analysis results, covariate balance before and after IPW, subgroup-stratified analyses by age, sex, and education, E-value analyses, alternative wave-definition sensitivity analyses, survey-weighted sensitivity analysis for HRS, denture status analyses in LASI, machine learning model performance metrics, SHAP feature importance rankings, Cox proportional hazards assumption tests based on Schoenfeld residuals, and propensity score model diagnostics, a lagged (staggered-wave) counterfactual mediation sensitivity analysis, and the covariate sets used in each analysis model. Supplementary figures include the Love plot of covariate balance before and after inverse probability weighting, machine learning model performance across cohorts and outcomes, SHAP feature importance heatmaps for ADL and IADL disability prediction, and an overview of the four-stage analytic pipeline.
(DOCX)
