# Supplementary material for: Edentulism and functional disability in older adults: A longitudinal and cross-national study in England, the United States, and India
Source: PLoS One. 2026 Oct 30;21(10):e0361422. doi: 10.1371/journal.pone.0361422 (PMC13722177; doi:10.1371/journal.pone.0361422)
Supplement: Supplementary file 1 [file pone.0361422.s001.docx]

**S1 Checklist. STROBE statement—checklist of items that should be included in reports of cohort/cross-sectional observational studies.**

*Manuscript: Edentulism and functional disability in older adults: a longitudinal and cross-national study in England, the United States, and India.*

| **Section / topic** | **Item no.** | **Recommendation** | **Reported on / in** |
| --- | --- | --- | --- |
| Title and abstract | 1 | (a) Indicate the study’s design with a commonly used term in the title or the abstract. (b) Provide in the abstract an informative and balanced summary of what was done and found. | Title page; Abstract |
| **Introduction** |  |  |  |
| Background / rationale | 2 | Explain the scientific background and rationale for the investigation being reported. | Introduction, paragraphs 1–4 |
| Objectives | 3 | State specific objectives, including any prespecified hypotheses. | Introduction, last paragraph |
| **Methods** |  |  |  |
| Study design | 4 | Present key elements of study design early in the paper. | Methods, “Study design and data sources” |
| Setting | 5 | Describe the setting, locations, and relevant dates, including periods of recruitment, exposure, follow-up, and data collection. | Methods, “Study design and data sources” |
| Participants | 6 | (a) Give the eligibility criteria, and the sources and methods of selection of participants. Describe methods of follow-up (cohort). (b) Report numbers of exposed and unexposed (cohort). | Methods, “Study design and data sources”; Results, “Sample characteristics”; Fig 2 |
| Variables | 7 | Clearly define all outcomes, exposures, predictors, potential confounders, and effect modifiers. Give diagnostic criteria, if applicable. | Methods, “Exposure, outcomes, mediators, and covariates” |
| Data sources / measurement | 8 | For each variable of interest, give sources of data and details of methods of assessment (measurement). Describe comparability of assessment methods if there is more than one group. | Methods, “Exposure, outcomes, mediators, and covariates”; Supplementary Materials |
| Bias | 9 | Describe any efforts to address potential sources of bias. | Methods, “Propensity score estimation and diagnostics” and “Sensitivity analyses” |
| Study size | 10 | Explain how the study size was arrived at. | Methods, “Study design and data sources”; sample sizes dictated by cohort availability |
| Quantitative variables | 11 | Explain how quantitative variables were handled in the analyses. If applicable, describe which groupings were chosen and why. | Methods, “Exposure, outcomes, mediators, and covariates” and “Data preprocessing” |
| Statistical methods | 12 | (a) Describe all statistical methods, including those used to control for confounding. (b) Describe any methods used to examine subgroups and interactions. (c) Explain how missing data were addressed. (d) If applicable, describe analytical methods taking account of sampling strategy. (e) Describe any sensitivity analyses. | Methods, “Statistical analysis”, “Longitudinal analysis and Cox model assumptions”, “Mediation analysis”, “Sensitivity analyses”; Supplementary Materials; Supplementary Table S3 |
| **Results** |  |  |  |
| Participants | 13 | (a) Report numbers of individuals at each stage of study. (b) Give reasons for non-participation at each stage. (c) Consider use of a flow diagram. | Fig 2; Results, “Sample characteristics” |
| Descriptive data | 14 | (a) Give characteristics of study participants and information on exposures and potential confounders. (b) Indicate number of participants with missing data for each variable of interest. (c) Summarize follow-up time (cohort). | Table 1; Methods, “Data preprocessing”; Results “Longitudinal associations” |
| Outcome data | 15 | Report numbers of outcome events or summary measures over time. | Results, “Sample characteristics” and “Longitudinal associations” |
| Main results | 16 | (a) Give unadjusted estimates and, if applicable, confounder-adjusted estimates and their precision (e.g., 95% CI). Make clear which confounders were adjusted for and why they were included. (b) Report category boundaries when continuous variables were categorized. (c) If relevant, consider translating estimates of relative risk into absolute risk for a meaningful time period. | Table 2; Fig 3; Fig 4; Results, “Cross-sectional associations” and “Longitudinal associations” |
| Other analyses | 17 | Report other analyses done (e.g., analyses of subgroups and interactions, and sensitivity analyses). | Results, “Mediation analyses” and “Sensitivity and supportive analyses”; Table 3; Fig 5; Fig 6; Supplementary Materials (Supplementary Tables S1–S11) |
| **Discussion** |  |  |  |
| Key results | 18 | Summarize key results with reference to study objectives. | Discussion, paragraph 1 |
| Limitations | 19 | Discuss limitations of the study, taking into account sources of potential bias or imprecision. Discuss both direction and magnitude of any potential bias. | Discussion, paragraph on limitations |
| Interpretation | 20 | Give a cautious overall interpretation of results considering objectives, limitations, multiplicity of analyses, results from similar studies, and other relevant evidence. | Discussion, paragraphs 2–8 |
| Generalizability | 21 | Discuss the generalizability (external validity) of the study results. | Discussion, paragraphs on LASI differences and practical implication |
| **Other information** |  |  |  |
| Funding | 22 | Give the source of funding and the role of the funders for the present study and, if applicable, for the original study on which the present article is based. | Financial disclosure statement submitted in the online system (per PLOS ONE policy); the authors received no specific funding for this work |

*Note: An Explanation and Elaboration article discusses each checklist item and gives methodological background and published examples of transparent reporting. The STROBE checklist is best used in conjunction with this article (freely available on the websites of PLOS Medicine at http://www.plosmedicine.org/, Annals of Internal Medicine at http://www.annals.org/, and Epidemiology at http://www.epidem.com/). Information on the STROBE Initiative is available at www.strobe-statement.org.*
