# Supplementary material for: Edentulism and functional disability in older adults: A longitudinal and cross-national study in England, the United States, and India
Source: PLoS One. 2026 Oct 30;21(10):e0361422. doi: 10.1371/journal.pone.0361422 (PMC13722177; doi:10.1371/journal.pone.0361422)
Supplement: Supplementary file 2 [file pone.0361422.s002.docx]

**Supplementary Materials**

*Edentulism and Functional Disability in Older Adults: A Longitudinal and Cross-National Study in England, the US, and India*

**Supplementary Methods**

**Multiple Imputation Procedure**

To assess the sensitivity of results to the imputation strategy, we conducted multiple imputation using five imputed datasets. For each dataset, missing values were imputed using an iterative random-forest-based imputer (missForest-style approach) with the same variable set as the primary single-imputation analysis. The primary IPW analysis was then repeated on each imputed dataset, and point estimates and variance components were pooled using Rubin’s rules. This procedure accounts for both within-imputation and between-imputation variability. Complete-case analysis was also performed as an additional benchmark.

**Propensity Score Model Details**

Propensity scores for edentulism were estimated using logistic regression with the following covariates: age, sex, education, smoking status, drinking status, hypertension, diabetes, heart disease, stroke, arthritis, lung disease, cancer, and recent falls. No interaction terms or nonlinear transformations were included. Stabilized inverse probability weights were calculated as w = P(A) / P(A|X) for the exposed and w = (1–P(A)) / (1–P(A|X)) for the unexposed, with trimming at the 1st and 99th percentiles of the propensity score distribution.

**Cox Proportional Hazards Assumption Testing**

The proportional hazards assumption was tested for each covariate using scaled Schoenfeld residuals. For the primary exposure variable (edentulism), the assumption was satisfied in all four Cox models: ELSA ADL (P = 0.99), ELSA IADL (P = 0.45), HRS ADL (P = 0.89), and HRS IADL (P = 0.54). Some time-varying effects were observed for age and arthritis, which is expected given the long follow-up period (median 6–8 years). Full results are shown below.

**Machine Learning Models**

Five prediction models were compared for each outcome (ADL disability, IADL disability) in each cohort (ELSA, HRS, LASI): logistic regression, random forest, ExtraTrees, XGBoost, and LightGBM. All models used the same covariate set as the main analysis. Hyperparameters were tuned using 5-fold cross-validation within the training set (80% of data), and performance was evaluated on the held-out test set (20%). Performance metrics included the area under the receiver operating characteristic curve (AUC-ROC), Brier score, and average precision. SHAP (SHapley Additive exPlanations) values were computed for the best-performing model in each cohort–outcome combination to rank feature importance.

**Supplementary Tables**

**S1 Table. Multiple imputation sensitivity analysis results for ADL and IADL disability.**

*Comparison of IPW odds ratios from single imputation, multiple imputation (5 datasets, Rubin’s rules), and complete-case analysis.*

**Panel A: ADL Disability**

| **Cohort** | **Method** | **OR** | **95% CI Low** | **95% CI High** | **N** |
| --- | --- | --- | --- | --- | --- |
| ELSA | Imputed | 1.30 | 1.17 | 1.46 | 9,102 |
| ELSA | Complete Case | 1.38 | 1.22 | 1.54 | 8,079 |
| ELSA | Multiple Imputation (x5) | 1.31 | 1.17 | 1.46 | 9,102 |
| HRS | Imputed | 1.23 | 1.15 | 1.31 | 22,156 |
| HRS | Complete Case | 1.23 | 1.14 | 1.31 | 19,297 |
| HRS | Multiple Imputation (x5) | 1.23 | 1.16 | 1.32 | 22,156 |
| LASI | Imputed | 1.40 | 1.29 | 1.51 | 31,816 |
| LASI | Complete Case | 1.40 | 1.29 | 1.52 | 31,573 |
| LASI | Multiple Imputation (x5) | 1.40 | 1.29 | 1.51 | 31,816 |

**Panel B: IADL Disability**

| **Cohort** | **Method** | **OR** | **95% CI Low** | **95% CI High** | **N** |
| --- | --- | --- | --- | --- | --- |
| ELSA | Imputed | 1.41 | 1.26 | 1.58 | 9,102 |
| ELSA | Complete Case | 1.48 | 1.32 | 1.67 | 8,079 |
| ELSA | Multiple Imputation (x5) | 1.41 | 1.26 | 1.58 | 9,102 |
| HRS | Imputed | 1.31 | 1.23 | 1.40 | 22,156 |
| HRS | Complete Case | 1.30 | 1.21 | 1.39 | 19,296 |
| HRS | Multiple Imputation (x5) | 1.32 | 1.23 | 1.40 | 22,156 |
| LASI | Imputed | 1.12 | 1.04 | 1.20 | 31,816 |
| LASI | Complete Case | 1.11 | 1.04 | 1.20 | 31,573 |
| LASI | Multiple Imputation (x5) | 1.11 | 1.04 | 1.20 | 31,816 |

**S2 Table. Covariate balance before and after inverse probability weighting.**

*Absolute standardized mean differences (SMD) for all covariates. All post-weighting SMDs were below 0.05.*

| **Variable** | **SMD (Raw)** | **SMD (Weighted)** | **Cohort** |
| --- | --- | --- | --- |
| Age | 0.609 | 0.007 | ELSA |
| Male sex | -0.166 | 0.006 | ELSA |
| Education | -0.731 | -0.022 | ELSA |
| Married/partnered | -0.403 | -0.010 | ELSA |
| Current smoker | 0.237 | 0.030 | ELSA |
| Current drinker | -0.368 | -0.028 | ELSA |
| Hypertension | 0.128 | 0.018 | ELSA |
| Diabetes | 0.129 | 0.013 | ELSA |
| Heart disease | 0.126 | 0.009 | ELSA |
| Stroke | 0.222 | 0.013 | ELSA |
| Arthritis | 0.170 | 0.003 | ELSA |
| Lung disease | 0.226 | 0.041 | ELSA |
| Cancer | -0.017 | -0.016 | ELSA |
| Fall history | 0.192 | -0.008 | ELSA |
| Age | 0.189 | 0.003 | HRS |
| Male sex | -0.018 | 0.001 | HRS |
| Education | -0.653 | -0.038 | HRS |
| Married/partnered | -0.277 | -0.010 | HRS |
| Current smoker | 0.291 | 0.008 | HRS |
| Current drinker | -0.364 | -0.016 | HRS |
| Hypertension | 0.163 | 0.026 | HRS |
| Diabetes | 0.119 | 0.027 | HRS |
| Heart disease | 0.193 | 0.012 | HRS |
| Stroke | 0.202 | 0.006 | HRS |
| Arthritis | 0.151 | -0.004 | HRS |
| Lung disease | 0.326 | 0.018 | HRS |
| Cancer | 0.012 | -0.010 | HRS |
| Fall history | 0.152 | 0.011 | HRS |
| Age | 0.599 | 0.024 | LASI |
| Male sex | -0.092 | -0.003 | LASI |
| Education | -0.108 | -0.015 | LASI |
| Married/partnered | -0.281 | -0.009 | LASI |
| Current smoker | 0.093 | -0.005 | LASI |
| Current drinker | -0.068 | -0.001 | LASI |
| Hypertension | 0.024 | -0.007 | LASI |
| Diabetes | -0.009 | -0.006 | LASI |
| Heart disease | 0.047 | 0.011 | LASI |
| Stroke | 0.026 | 0.006 | LASI |
| Arthritis | -0.025 | -0.011 | LASI |
| Lung disease | 0.031 | 0.006 | LASI |
| Cancer | 0.023 | 0.017 | LASI |
| Fall history | 0.088 | -0.003 | LASI |

**S3 Table. Subgroup stratified analysis results.**

*IPW odds ratios stratified by age group, sex, education level, and comorbidity burden.*

**Panel A: ADL Disability**

| **Cohort** | **Stratification** | **Stratum** | **OR** | **CI Low** | **CI High** | **P** | **P interaction** | **N** |
| --- | --- | --- | --- | --- | --- | --- | --- | --- |
| ELSA | Sex | Female | 1.40 | 1.21 | 1.61 | <0.001 | 0.067 | 4,893 |
| ELSA | Sex | Male | 1.20 | 1.01 | 1.43 | 0.041 | 0.067 | 4,209 |
| HRS | Sex | Female | 1.27 | 1.17 | 1.39 | <0.001 | 0.060 | 12,667 |
| HRS | Sex | Male | 1.18 | 1.07 | 1.31 | 0.001 | 0.060 | 9,489 |
| LASI | Sex | Female | 1.30 | 1.17 | 1.44 | <0.001 | 0.119 | 16,532 |
| LASI | Sex | Male | 1.55 | 1.37 | 1.75 | <0.001 | 0.119 | 15,284 |
| ELSA | Age Group | 60-69 | 1.73 | 1.21 | 2.49 | 0.003 | 0.121 | 1,772 |
| ELSA | Age Group | 70-79 | 1.32 | 1.09 | 1.60 | 0.005 | 0.121 | 4,077 |
| ELSA | Age Group | 80+ | 1.14 | 0.98 | 1.33 | 0.082 | 0.121 | 3,253 |
| HRS | Age Group | 60-69 | 1.31 | 1.13 | 1.51 | <0.001 | 0.002 | 6,719 |
| HRS | Age Group | 70-79 | 1.29 | 1.14 | 1.46 | <0.001 | 0.002 | 7,055 |
| HRS | Age Group | 80+ | 1.13 | 1.03 | 1.25 | 0.009 | 0.002 | 8,382 |
| LASI | Age Group | 60-69 | 1.41 | 1.23 | 1.62 | <0.001 | 0.258 | 19,160 |
| LASI | Age Group | 70-79 | 1.32 | 1.17 | 1.50 | <0.001 | 0.258 | 9,227 |
| LASI | Age Group | 80+ | 1.59 | 1.36 | 1.85 | <0.001 | 0.258 | 3,429 |
| ELSA | Education | Low | 1.29 | 1.12 | 1.49 | <0.001 | 0.360 | 3,731 |
| ELSA | Education | Medium | 1.23 | 1.00 | 1.50 | 0.051 | 0.360 | 4,168 |
| ELSA | Education | High | 1.30 | 0.72 | 2.36 | 0.385 | 0.360 | 1,203 |
| HRS | Education | Low | 1.18 | 1.05 | 1.32 | 0.005 | 0.754 | 4,996 |
| HRS | Education | Medium | 1.22 | 1.12 | 1.33 | <0.001 | 0.754 | 12,610 |
| HRS | Education | High | 1.36 | 1.07 | 1.74 | 0.013 | 0.754 | 4,550 |
| LASI | Education | Low | 1.41 | 1.29 | 1.54 | <0.001 | 0.688 | 24,784 |
| LASI | Education | Medium | 1.45 | 1.17 | 1.80 | <0.001 | 0.688 | 5,722 |
| LASI | Education | High | 0.96 | 0.53 | 1.73 | 0.885 | 0.688 | 1,310 |

**Panel B: IADL Disability**

| **Cohort** | **Stratification** | **Stratum** | **OR** | **CI Low** | **CI High** | **P** | **P interaction** | **N** |
| --- | --- | --- | --- | --- | --- | --- | --- | --- |
| ELSA | Sex | Female | 1.33 | 1.15 | 1.54 | <0.001 | 0.903 | 4,893 |
| ELSA | Sex | Male | 1.49 | 1.24 | 1.79 | <0.001 | 0.903 | 4,209 |
| HRS | Sex | Female | 1.30 | 1.20 | 1.41 | <0.001 | 0.679 | 12,667 |
| HRS | Sex | Male | 1.33 | 1.20 | 1.48 | <0.001 | 0.679 | 9,489 |
| LASI | Sex | Female | 1.09 | 0.99 | 1.20 | 0.066 | 0.172 | 16,532 |
| LASI | Sex | Male | 1.16 | 1.04 | 1.30 | 0.007 | 0.172 | 15,284 |
| ELSA | Age Group | 60-69 | 1.81 | 1.20 | 2.72 | 0.004 | 0.900 | 1,772 |
| ELSA | Age Group | 70-79 | 1.34 | 1.07 | 1.67 | 0.009 | 0.900 | 4,077 |
| ELSA | Age Group | 80+ | 1.35 | 1.16 | 1.57 | <0.001 | 0.900 | 3,253 |
| HRS | Age Group | 60-69 | 1.31 | 1.12 | 1.52 | <0.001 | 0.229 | 6,719 |
| HRS | Age Group | 70-79 | 1.31 | 1.16 | 1.48 | <0.001 | 0.229 | 7,055 |
| HRS | Age Group | 80+ | 1.32 | 1.20 | 1.45 | <0.001 | 0.229 | 8,382 |
| LASI | Age Group | 60-69 | 1.03 | 0.92 | 1.17 | 0.572 | 0.001 | 19,160 |
| LASI | Age Group | 70-79 | 1.13 | 1.01 | 1.27 | 0.034 | 0.001 | 9,227 |
| LASI | Age Group | 80+ | 1.44 | 1.22 | 1.69 | <0.001 | 0.001 | 3,429 |
| ELSA | Education | Low | 1.39 | 1.20 | 1.60 | <0.001 | 0.529 | 3,731 |
| ELSA | Education | Medium | 1.31 | 1.05 | 1.63 | 0.016 | 0.529 | 4,168 |
| ELSA | Education | High | 1.56 | 0.81 | 3.00 | 0.186 | 0.529 | 1,203 |
| HRS | Education | Low | 1.19 | 1.06 | 1.33 | 0.002 | 0.219 | 4,996 |
| HRS | Education | Medium | 1.31 | 1.20 | 1.43 | <0.001 | 0.219 | 12,610 |
| HRS | Education | High | 1.59 | 1.25 | 2.02 | <0.001 | 0.219 | 4,550 |
| LASI | Education | Low | 1.12 | 1.04 | 1.21 | 0.004 | 0.401 | 24,784 |
| LASI | Education | Medium | 1.11 | 0.90 | 1.36 | 0.332 | 0.401 | 5,722 |
| LASI | Education | High | 0.85 | 0.48 | 1.52 | 0.592 | 0.401 | 1,310 |

**S4 Table. E-value analysis results.**

*E-values indicating the minimum strength of association an unmeasured confounder would need to explain away the observed association.*

**Panel A: ADL Disability**

| **Cohort** | **OR** | **CI Low** | **CI High** | **E-value** | **E-value CI** |
| --- | --- | --- | --- | --- | --- |
| ELSA | 1.30 | 1.17 | 1.46 | 1.93 | 1.61 |
| HRS | 1.23 | 1.15 | 1.31 | 1.76 | 1.57 |
| LASI | 1.40 | 1.29 | 1.51 | 2.14 | 1.90 |

**Panel B: IADL Disability**

| **Cohort** | **OR** | **CI Low** | **CI High** | **E-value** | **E-value CI** |
| --- | --- | --- | --- | --- | --- |
| ELSA | 1.41 | 1.26 | 1.58 | 2.18 | 1.83 |
| HRS | 1.31 | 1.23 | 1.40 | 1.95 | 1.76 |
| LASI | 1.12 | 1.04 | 1.20 | 1.47 | 1.24 |

**S5 Table. Wave definition sensitivity analysis.**

*IPW odds ratios under alternative cross-sectional snapshot definitions.*

**Panel A: ADL Disability**

| **Scenario** | **Cohort** | **OR** | **CI Low** | **CI High** | **P** | **N** |
| --- | --- | --- | --- | --- | --- | --- |
| Main: last snapshot + latest exposure | ELSA | 1.30 | 1.17 | 1.46 | <0.001 | 9,102 |
| Main: last snapshot + latest exposure | HRS | 1.23 | 1.15 | 1.31 | <0.001 | 22,156 |
| Main: last snapshot + latest exposure | LASI | 1.40 | 1.29 | 1.51 | <0.001 | 31,816 |
| First eligible snapshot | ELSA | 1.21 | 1.06 | 1.38 | 0.006 | 9,102 |
| First eligible snapshot | HRS | 1.15 | 1.05 | 1.26 | 0.002 | 22,156 |
| First eligible snapshot | LASI | 1.40 | 1.29 | 1.51 | <0.001 | 31,816 |
| Last snapshot + baseline exposure | ELSA | 1.31 | 1.18 | 1.47 | <0.001 | 9,102 |
| Last snapshot + baseline exposure | HRS | 1.24 | 1.16 | 1.33 | <0.001 | 22,156 |
| Last snapshot + baseline exposure | LASI | 1.40 | 1.29 | 1.51 | <0.001 | 31,816 |

**Panel B: IADL Disability**

| **Scenario** | **Cohort** | **OR** | **CI Low** | **CI High** | **P** | **N** |
| --- | --- | --- | --- | --- | --- | --- |
| Main: last snapshot + latest exposure | ELSA | 1.41 | 1.26 | 1.58 | <0.001 | 9,102 |
| Main: last snapshot + latest exposure | HRS | 1.31 | 1.23 | 1.40 | <0.001 | 22,156 |
| Main: last snapshot + latest exposure | LASI | 1.12 | 1.04 | 1.20 | 0.003 | 31,816 |
| First eligible snapshot | ELSA | 1.47 | 1.25 | 1.72 | <0.001 | 9,102 |
| First eligible snapshot | HRS | 1.30 | 1.18 | 1.44 | <0.001 | 22,156 |
| First eligible snapshot | LASI | 1.12 | 1.04 | 1.20 | 0.003 | 31,816 |
| Last snapshot + baseline exposure | ELSA | 1.45 | 1.29 | 1.63 | <0.001 | 9,102 |
| Last snapshot + baseline exposure | HRS | 1.34 | 1.25 | 1.44 | <0.001 | 22,156 |
| Last snapshot + baseline exposure | LASI | 1.12 | 1.04 | 1.20 | 0.003 | 31,816 |

**S6 Table. Survey-weighted sensitivity analysis (HRS).**

*IPW odds ratios using person-level survey weights to account for the complex sampling design of HRS.*

| **Outcome** | **Cohort** | **Method** | **OR** | **CI Low** | **CI High** | **P** | **N** |
| --- | --- | --- | --- | --- | --- | --- | --- |
| ADL | HRS | Survey-weighted IPW | 1.31 | 1.21 | 1.42 | <0.001 | 20,628 |
| IADL | HRS | Survey-weighted IPW | 1.44 | 1.33 | 1.56 | <0.001 | 20,628 |

**S7 Table. Denture status analysis (LASI).**

*Odds ratios comparing edentulous participants with and without dentures against non-edentulous participants.*

**Panel A: ADL Disability**

| **Cohort** | **Comparison** | **OR** | **CI low** | **CI high** | **P** | **N** | **N, non-edentulous** | **N, edentulous without denture** | **N, edentulous with denture** |
| --- | --- | --- | --- | --- | --- | --- | --- | --- | --- |
| LASI | Edentulous without denture vs non-edentulous | 1.52 | 1.39 | 1.66 | <0.001 | 31,774 | 28,214 | 2,875 | 685 |
| LASI | Edentulous with denture vs non-edentulous | 1.02 | 0.85 | 1.23 | 0.816 | 31,774 | 28,214 | 2,875 | 685 |
| LASI | Denture use vs no denture among edentulous | 0.66 | 0.54 | 0.82 | <0.001 | 3,560 |  | 2,875 | 685 |

**Panel B: IADL Disability**

| **Cohort** | **Comparison** | **OR** | **CI low** | **CI high** | **P** | **N** | **N, non-edentulous** | **N, edentulous without denture** | **N, edentulous with denture** |
| --- | --- | --- | --- | --- | --- | --- | --- | --- | --- |
| LASI | Edentulous without denture vs non-edentulous | 1.26 | 1.16 | 1.37 | <0.001 | 31,774 | 28,214 | 2,875 | 685 |
| LASI | Edentulous with denture vs non-edentulous | 0.83 | 0.70 | 0.98 | 0.031 | 31,774 | 28,214 | 2,875 | 685 |
| LASI | Denture use vs no denture among edentulous | 0.65 | 0.53 | 0.78 | <0.001 | 3,560 |  | 2,875 | 685 |

**S8 Table. Machine learning model performance.**

*AUC-ROC, Brier score, and average precision for five prediction models across all cohort–outcome combinations.*

| **Cohort** | **Outcome** | **Model** | **AUC** | **Brier** | **AP** | **N** |
| --- | --- | --- | --- | --- | --- | --- |
| ELSA | ADL | Logistic | 0.841 | 0.141 | 0.672 | 9,102 |
| ELSA | ADL | RF | 0.873 | 0.127 | 0.766 | 9,102 |
| ELSA | ADL | ExtraTrees | 0.844 | 0.141 | 0.696 | 9,102 |
| ELSA | ADL | XGBoost | 0.885 | 0.120 | 0.781 | 9,102 |
| ELSA | ADL | LightGBM | 0.885 | 0.120 | 0.781 | 9,102 |
| HRS | ADL | Logistic | 0.861 | 0.134 | 0.739 | 22,156 |
| HRS | ADL | RF | 0.898 | 0.116 | 0.829 | 22,156 |
| HRS | ADL | ExtraTrees | 0.866 | 0.135 | 0.763 | 22,156 |
| HRS | ADL | XGBoost | 0.916 | 0.104 | 0.856 | 22,156 |
| HRS | ADL | LightGBM | 0.917 | 0.104 | 0.856 | 22,156 |
| LASI | ADL | Logistic | 0.717 | 0.147 | 0.422 | 31,816 |
| LASI | ADL | RF | 0.722 | 0.145 | 0.438 | 31,816 |
| LASI | ADL | ExtraTrees | 0.711 | 0.148 | 0.410 | 31,816 |
| LASI | ADL | XGBoost | 0.722 | 0.144 | 0.457 | 31,816 |
| LASI | ADL | LightGBM | 0.724 | 0.143 | 0.459 | 31,816 |
| ELSA | IADL | Logistic | 0.887 | 0.108 | 0.732 | 9,102 |
| ELSA | IADL | RF | 0.916 | 0.096 | 0.819 | 9,102 |
| ELSA | IADL | ExtraTrees | 0.897 | 0.108 | 0.766 | 9,102 |
| ELSA | IADL | XGBoost | 0.925 | 0.088 | 0.835 | 9,102 |
| ELSA | IADL | LightGBM | 0.927 | 0.087 | 0.838 | 9,102 |
| HRS | IADL | Logistic | 0.875 | 0.127 | 0.768 | 22,156 |
| HRS | IADL | RF | 0.907 | 0.109 | 0.852 | 22,156 |
| HRS | IADL | ExtraTrees | 0.879 | 0.129 | 0.790 | 22,156 |
| HRS | IADL | XGBoost | 0.922 | 0.098 | 0.873 | 22,156 |
| HRS | IADL | LightGBM | 0.923 | 0.098 | 0.874 | 22,156 |
| LASI | IADL | Logistic | 0.723 | 0.204 | 0.627 | 31,816 |
| LASI | IADL | RF | 0.731 | 0.202 | 0.638 | 31,816 |
| LASI | IADL | ExtraTrees | 0.718 | 0.206 | 0.618 | 31,816 |
| LASI | IADL | XGBoost | 0.732 | 0.201 | 0.642 | 31,816 |
| LASI | IADL | LightGBM | 0.734 | 0.200 | 0.646 | 31,816 |

**S9 Table. SHAP feature importance rankings.**

*Top features ranked by mean absolute SHAP value for the best-performing model.*

**Panel A: ADL Disability**

| **Feature** | **ELSA importance** | **ELSA rank** | **HRS importance** | **HRS rank** | **LASI importance** | **LASI rank** | **Mean rank** |
| --- | --- | --- | --- | --- | --- | --- | --- |
| Grip strength | 0.9348 | 1 | 0.8100 | 2 | 0.3239 | 1 | 1.33 |
| Depressive symptoms | 0.6819 | 2 | 0.8116 | 1 | 0.2735 | 2 | 1.67 |
| Age | 0.2262 | 5 | 0.2106 | 6 | 0.2458 | 3 | 4.67 |
| BMI | 0.2809 | 4 | 0.5036 | 3 | 0.0742 | 7 | 4.67 |
| Male sex | 0.6529 | 3 | 0.3964 | 4 | 0.0489 | 10 | 5.67 |
| Fall history | 0.0920 | 8 | 0.2757 | 5 | 0.1399 | 5 | 6.00 |
| Arthritis | 0.2159 | 6 | 0.1515 | 8 | 0.1737 | 4 | 6.00 |
| Stroke | 0.0865 | 10 | 0.1318 | 9 | 0.0533 | 9 | 9.33 |
| Hypertension | 0.0292 | 14 | 0.0756 | 13 | 0.0943 | 6 | 11.00 |
| Diabetes | 0.0354 | 13 | 0.1724 | 7 | 0.0250 | 13 | 11.00 |
| Lung disease | 0.1250 | 7 | 0.0847 | 11 | 0.0008 | 18 | 12.00 |
| Education | 0.0876 | 9 | 0.0505 | 14 | 0.0421 | 11 | 11.33 |
| Married/partnered | 0.0235 | 16 | 0.0391 | 15 | 0.0081 | 15 | 15.33 |
| Heart disease | 0.0852 | 11 | 0.0374 | 16 | 0.0249 | 14 | 13.67 |
| Current drinker | 0.0259 | 15 | 0.0906 | 10 | 0.0036 | 17 | 14.00 |
| Current smoker | 0.0131 | 18 | 0.0765 | 12 | 0.0276 | 12 | 14.00 |
| Edentulism | 0.0165 | 17 | 0.0208 | 18 | 0.0551 | 8 | 14.33 |
| Cancer | 0.0479 | 12 | 0.0232 | 17 | 0.0058 | 16 | 15.00 |

**Panel B: IADL Disability**

| **Feature** | **ELSA importance** | **ELSA rank** | **HRS importance** | **HRS rank** | **LASI importance** | **LASI rank** | **Mean rank** |
| --- | --- | --- | --- | --- | --- | --- | --- |
| Grip strength | 1.3641 | 1 | 1.0321 | 1 | 0.3845 | 1 | 1.00 |
| Depressive symptoms | 0.6078 | 3 | 0.7904 | 2 | 0.2380 | 4 | 3.00 |
| Age | 0.2327 | 5 | 0.1961 | 5 | 0.2528 | 3 | 4.33 |
| Male sex | 0.9056 | 2 | 0.4618 | 3 | 0.0600 | 10 | 5.00 |
| BMI | 0.2387 | 4 | 0.1605 | 7 | 0.1067 | 5 | 5.33 |
| Education | 0.0835 | 10 | 0.0679 | 10 | 0.2812 | 2 | 7.33 |
| Married/partnered | 0.0295 | 16 | 0.0161 | 15 | 0.0706 | 8 | 13.00 |
| Fall history | 0.0663 | 11 | 0.2114 | 4 | 0.0931 | 7 | 7.33 |
| Stroke | 0.1277 | 6 | 0.0988 | 8 | 0.0453 | 11 | 8.33 |
| Arthritis | 0.1019 | 7 | 0.0109 | 16 | 0.1011 | 6 | 9.67 |
| Current drinker | 0.0644 | 12 | 0.1930 | 6 | 0.0228 | 12 | 10.00 |
| Lung disease | 0.0852 | 9 | 0.0986 | 9 | 0.0050 | 17 | 11.67 |
| Heart disease | 0.0977 | 8 | 0.0394 | 13 | 0.0082 | 15 | 12.00 |
| Edentulism | 0.0426 | 13 | 0.0617 | 11 | 0.0160 | 13 | 12.33 |
| Diabetes | 0.0375 | 14 | 0.0490 | 12 | 0.0070 | 16 | 14.00 |
| Hypertension | 0.0249 | 17 | 0.0108 | 17 | 0.0696 | 9 | 14.33 |
| Current smoker | 0.0223 | 18 | 0.0336 | 14 | 0.0115 | 14 | 15.33 |
| Cancer | 0.0331 | 15 | 0.0071 | 18 | 0.0005 | 18 | 17.00 |

**S10 Table. Cox proportional hazards assumption test (Schoenfeld residuals).**

*P values > 0.05 indicate the proportional hazards assumption is satisfied for that variable.*

| **Cohort** | **Outcome** | **Variable** | **Test statistic** | **P value** |
| --- | --- | --- | --- | --- |
| ELSA | ADL | Age | 12.2 | <0.001 |
| ELSA | ADL | Arthritis | 12.5 | <0.001 |
| ELSA | ADL | Cancer | 0.0667 | 0.796 |
| ELSA | ADL | Diabetes | 2.06 | 0.151 |
| ELSA | ADL | Current drinker | 0.64 | 0.424 |
| ELSA | ADL | Education | 0.527 | 0.468 |
| ELSA | ADL | Fall history | 0.00992 | 0.921 |
| ELSA | ADL | Male sex | 0.00157 | 0.968 |
| ELSA | ADL | Heart disease | 0.0895 | 0.765 |
| ELSA | ADL | Hypertension | 0.545 | 0.460 |
| ELSA | ADL | Lung disease | 0.0524 | 0.819 |
| ELSA | ADL | Married/partnered | 0.00433 | 0.948 |
| ELSA | ADL | Edentulism | 0.000545 | 0.981 |
| ELSA | ADL | Current smoker | 0.638 | 0.425 |
| ELSA | ADL | Stroke | 0.599 | 0.439 |
| ELSA | IADL | Age | 16.2 | <0.001 |
| ELSA | IADL | Arthritis | 5.37 | 0.021 |
| ELSA | IADL | Cancer | 2.17 | 0.141 |
| ELSA | IADL | Diabetes | 0.023 | 0.879 |
| ELSA | IADL | Current drinker | 1.31 | 0.252 |
| ELSA | IADL | Education | 0.581 | 0.446 |
| ELSA | IADL | Fall history | 0.0344 | 0.853 |
| ELSA | IADL | Male sex | 0.714 | 0.398 |
| ELSA | IADL | Heart disease | 0.694 | 0.405 |
| ELSA | IADL | Hypertension | 0.148 | 0.700 |
| ELSA | IADL | Lung disease | 0.0394 | 0.843 |
| ELSA | IADL | Married/partnered | 0.687 | 0.407 |
| ELSA | IADL | Edentulism | 0.54 | 0.463 |
| ELSA | IADL | Current smoker | 0.0727 | 0.788 |
| ELSA | IADL | Stroke | 2.08 | 0.149 |
| HRS | ADL | Age | 35.2 | <0.001 |
| HRS | ADL | Arthritis | 13 | <0.001 |
| HRS | ADL | Cancer | 1.32 | 0.251 |
| HRS | ADL | Diabetes | 0.00238 | 0.961 |
| HRS | ADL | Current drinker | 1.06 | 0.302 |
| HRS | ADL | Education | 4.44 | 0.035 |
| HRS | ADL | Fall history | 0.319 | 0.572 |
| HRS | ADL | Male sex | 0.111 | 0.739 |
| HRS | ADL | Heart disease | 0.102 | 0.749 |
| HRS | ADL | Hypertension | 0.393 | 0.531 |
| HRS | ADL | Lung disease | 5.72 | 0.017 |
| HRS | ADL | Married/partnered | 4.89 | 0.027 |
| HRS | ADL | Edentulism | 0.07 | 0.791 |
| HRS | ADL | Current smoker | 10 | 0.002 |
| HRS | ADL | Stroke | 4.35 | 0.037 |
| HRS | IADL | Age | 27.3 | <0.001 |
| HRS | IADL | Arthritis | 0.675 | 0.411 |
| HRS | IADL | Cancer | 0.843 | 0.358 |
| HRS | IADL | Diabetes | 1.95 | 0.162 |
| HRS | IADL | Current drinker | 4.75 | 0.029 |
| HRS | IADL | Education | 10.4 | 0.001 |
| HRS | IADL | Fall history | 0.312 | 0.577 |
| HRS | IADL | Male sex | 0.0748 | 0.785 |
| HRS | IADL | Heart disease | 1.21 | 0.271 |
| HRS | IADL | Hypertension | 0.126 | 0.722 |
| HRS | IADL | Lung disease | 0.63 | 0.427 |
| HRS | IADL | Married/partnered | 7.21 | 0.007 |
| HRS | IADL | Edentulism | 0.221 | 0.638 |
| HRS | IADL | Current smoker | 1.33 | 0.248 |
| HRS | IADL | Stroke | 1.36 | 0.243 |

**S11 Table. Propensity score model diagnostics.**

*C-statistic, weight distribution, and extreme weight percentages for each cohort.*

| **C-statistic** | **PS, mean** | **PS, median** | **PS, minimum** | **PS, maximum** | **Weight, mean** | **Weight, median** | **Weight, minimum** | **Weight, maximum** | **Weight, 1st percentile** | **Weight, 99th percentile** | **Weight, %>10** | **N** | **Cohort** |
| --- | --- | --- | --- | --- | --- | --- | --- | --- | --- | --- | --- | --- | --- |
| 0.762 | 0.2004 | 0.1544 | 0.0119 | 0.8037 | 0.9972 | 0.9033 | 0.2493 | 13.54 | 0.3365 | 2.78 | 0.0220 | 9,102 | ELSA |
| 0.727 | 0.2508 | 0.2187 | 0.0372 | 0.8469 | 0.9973 | 0.9111 | 0.2962 | 6.75 | 0.3893 | 2.64 | 0.0000 | 22,156 | HRS |
| 0.677 | 0.1119 | 0.0890 | 0.0341 | 0.7357 | 0.9993 | 0.9708 | 0.1551 | 3.36 | 0.3778 | 1.84 | 0.0000 | 31,816 | LASI |

**Supplementary Figures**

**S1 Fig. Love plot showing covariate balance before and after inverse probability weighting.**

*Absolute standardized mean differences for all covariates in ELSA, HRS, and LASI. The dashed line indicates the 0.10 threshold for adequate balance.*

**S2 Fig. Machine learning model performance comparison.**

*AUC-ROC values for five prediction models across all cohort–outcome combinations. LightGBM achieved the highest discrimination in all settings.*

**S3 Fig. SHAP heatmaps for ADL and IADL disability prediction.**

*Features are ranked by mean absolute SHAP value. Grip strength, depressive symptoms, and age consistently ranked among the top predictors.*

**(a) ADL Disability**

**(b) IADL Disability**

**S4 Fig. Overview of the analytic pipeline.**

The four pre-specified stages of the analysis (cross-sectional weighting with IPW and AIPW; longitudinal Cox modelling of temporal ordering; mediation analysis of grip strength, BMI, and depressive symptoms; and sensitivity and supportive machine-learning analyses), together with the cohorts and outcomes entering each stage.

## Supplementary Table S12. Lagged (staggered-wave) counterfactual mediation sensitivity analysis.

| Cohort | Scheme | Outcome | Pathway | N | NIE (95% CI) | NDE (95% CI) | TE | PM (%) |
| --- | --- | --- | --- | --- | --- | --- | --- | --- |
| ELSA | w3->w4->w6 | ADL | Grip | 2,538 | 0.0017 (-0.0007, 0.0049) | -0.0029 (-0.0406, 0.0379) | -0.0012 | -0.4 (-191.4, 179.9) |
| ELSA | w3->w4->w6 | ADL | BMI | 2,512 | 0.0058 (0.0021, 0.0107) | 0.0023 (-0.0351, 0.0449) | 0.0081 | 22.1 (-324.5, 549.2) |
| ELSA | w3->w4->w6 | ADL | Depression | 2,832 | -0.0020 (-0.0055, 0.0005) | 0.0081 (-0.0286, 0.0478) | 0.0061 | -5.0 (-114.7, 148.7) |
| ELSA | w3->w4->w6 | IADL | Grip | 2,538 | 0.0027 (-0.0010, 0.0070) | 0.0030 (-0.0274, 0.0368) | 0.0057 | 9.8 (-253.2, 328.0) |
| ELSA | w3->w4->w6 | IADL | BMI | 2,512 | 0.0006 (-0.0017, 0.0031) | 0.0102 (-0.0209, 0.0444) | 0.0108 | 1.9 (-60.1, 84.5) |
| ELSA | w3->w4->w6 | IADL | Depression | 2,832 | -0.0023 (-0.0060, 0.0008) | 0.0185 (-0.0117, 0.0518) | 0.0162 | -9.2 (-148.4, 120.2) |
| HRS | w8->w10->w12 | ADL | Grip | 2,514 | 0.0017 (-0.0022, 0.0060) | 0.0463 (0.0033, 0.0919) | 0.0480 | 3.3 (-8.5, 22.7) |
| HRS | w8->w10->w12 | ADL | BMI | 2,469 | 0.0013 (-0.0023, 0.0053) | 0.0438 (0.0008, 0.0905) | 0.0451 | 2.7 (-8.6, 22.1) |
| HRS | w8->w10->w12 | ADL | Depression | 5,761 | 0.0018 (-0.0015, 0.0054) | 0.0307 (0.0022, 0.0602) | 0.0325 | 5.5 (-7.5, 30.8) |
| HRS | w8->w10->w12 | IADL | Grip | 2,514 | 0.0018 (-0.0023, 0.0063) | 0.0592 (0.0147, 0.1066) | 0.0610 | 2.6 (-6.2, 15.2) |
| HRS | w8->w10->w12 | IADL | BMI | 2,469 | -0.0000 (-0.0012, 0.0012) | 0.0725 (0.0264, 0.1223) | 0.0725 | -0.0 (-2.1, 2.1) |
| HRS | w8->w10->w12 | IADL | Depression | 5,761 | 0.0021 (-0.0017, 0.0063) | 0.0389 (0.0107, 0.0683) | 0.0410 | 5.2 (-5.5, 23.4) |

Note: Exposure, mediator, and outcome measured at separate waves (ELSA w3→w4→w6, ≈6 y; HRS w8→w10→w12, ≈8 y). Effects on the risk-difference scale (R mediation package, counterfactual/g-computation, 1,000 simulations). LASI is cross-sectional and not included. PM% is a secondary descriptive measure and is unstable when the total effect is small.

## Supplementary Table S13. Covariate sets used in each analysis model.

| Model | Estimand / outcome | Covariates | Software / estimator |
| --- | --- | --- | --- |
| Propensity-score model | edentulism ~ X | age, gender, education, marital, smoking, drinking, hypertension, diabetes, heart, stroke, arthritis, lung, cancer, fall (14 variables) | scikit-learn LogisticRegression |
| AIPW outcome model | Y ~ A + X (per treatment arm) | age, gender, education, marital, smoking, drinking, hypertension, diabetes, heart, stroke, arthritis, lung, cancer, fall (14 variables) | scikit-learn LogisticRegression |
| Cox proportional-hazards | time-to-event Y ~ A + X | age, gender, education, marital, smoking, drinking, hypertension, diabetes, heart, stroke, arthritis, lung, cancer, fall (14 variables) | lifelines CoxPHFitter |
| Mediator model (A→M) | M ~ A + X | age, gender, education, marital, smoking, drinking, hypertension, diabetes, heart, stroke, arthritis, lung, cancer, fall (14 variables) | R mediation (lm / glm binomial) |
| Mediation outcome model (A+M→Y) | Y ~ A + M + X | age, gender, education, marital, smoking, drinking, hypertension, diabetes, heart, stroke, arthritis, lung, cancer, fall (14 variables) | R mediation (glm binomial; g-computation) |
| Machine-learning features | Y ~ edentulism + X + grip strength + BMI + depressive symptoms | age, gender, education, marital, smoking, drinking, hypertension, diabetes, heart, stroke, arthritis, lung, cancer, fall (14 variables) + grip strength, BMI, depressive symptoms | LightGBM/XGBoost/RF/ExtraTrees/Logistic/GAM |

Note: All models reference a single, unified 14-variable covariate list (age, sex, education, marital status, smoking, drinking, hypertension, diabetes, heart disease, stroke, arthritis, lung disease, cancer, and fall history). The covariate specifications in the Methods are now identical to this set. Marital status was recoded as 1 = married/partnered and 0 = other, with 0% missingness in all cohorts. No interaction terms were specified.
